# Rainfall-enhanced blooming in typhoon wakes

**DOI:** 10.1038/srep31310

**Published:** 2016-08-22

**Authors:** Y.-C. Lin, L.-Y. Oey

**Affiliations:** 1 Graduate Institute of Hydrological & Oceanic Sciences, National Central University, 300 Zhongda Rd., Jhongli City, Taoyuan 320, Taiwan; 2 Program in Atmospheric & Oceanic Sciences, 300 Forrestal Rd, Sayre Hall, Princeton University, Princeton, New Jersey, USA

## Abstract

Strong phytoplankton blooming in tropical-cyclone (TC) wakes over the oligotrophic oceans potentially contributes to long-term changes in global biogeochemical cycles. Yet blooming has traditionally been discussed using anecdotal events and its biophysical mechanics remain poorly understood. Here we identify dominant blooming patterns using 16 years of ocean-color data in the wakes of 141 typhoons in western North Pacific. We observe right-side asymmetric blooming shortly after the storms, attributed previously to sub-mesoscale re-stratification, but thereafter a left-side asymmetry which coincides with the left-side preference in rainfall due to the large-scale wind shear. Biophysical model experiments and observations demonstrate that heavier rainfall freshens the near-surface water, leading to stronger stratification, decreased turbulence and enhanced blooming. Our results suggest that rainfall plays a previously unrecognized, critical role in TC-induced blooming, with potentially important implications for global biogeochemical cycles especially in view of the recent and projected increases in TC-intensity that harbingers stronger mixing and heavier rain under the storm.

Through photosynthetic carbon fixation, phytoplankton removes CO_2_ from the atmosphere, and contributes to changes in global biogeochemical cycles and climate^1^. Due to climate warming^2^, strengthening ocean’s stratification in the tropics and subtropics has contributed to the long-term decline of global phytoplankton^3^,^4^. Mixing, upwelling and sub-mesoscale processes in tropical cyclone (TC) wakes^5^,^6^ bring nutrients to the euphotic surface layer of the ocean, and produce phytoplankton blooms which are often many times greater than by other causes^6^,^7^,^8^,^9^,^10^,^11^,^12^,^13^,^14^,^15^. Although the phenomenon is relatively short-lived (~10 days), given the TC locations over the oligotrophic tropical and subtropical ocean, TC-induced blooming may be a significant modulator of the long-term variability of the net primary production^16^. Despite its potential importance, descriptions of TC-induced blooming have largely been based on anecdotal events^6^,^7^,^8^,^9^,^10^,^11^,^12^,^13^,^14^,^15^. This work extends the previous studies and attempts to provide a comprehensive description and understanding of the blooming process by analyzing observations as well as modeling.

There are two generally accepted hypotheses of phytoplankton blooming. Gran and Braarud^17^ pointed out that blooming near the sea surface cannot occur if sufficiently strong turbulence spreads phytoplankton cells below the illuminated zone, so that destruction by respiration exceeds production by photosynthesis. There exists a critical depth *h*_*crit*_, such that after mixing, blooming occurs only if the mixed-layer depth MLD becomes less than h_crit_; this is known as the Sverdrup’s^18^ critical-depth hypothesis. Later studies show that blooming can occur in a deep mixed layer provided that turbulence is below a critical threshold, so that phytoplankton can stay in the well-lit surface layer and outgrow the vertical mixing rates^19^,^20^; this is known as the critical-turbulence hypothesis.

TC-induced blooming is similar to that of spring blooming after winter mixing, except for the different time scales and also that the cold core in the wake’s center forms double mixed-layer fronts on both sides. In the early phase^6^, 1~5 days after the TC, sub-meso scale recirculation cells develop, caused by symmetric instability of the mixed-layer fronts, but re-stratification occurs more rapidly on the right side (northern hemisphere) where turbulence decreases and strong blooming is observed (Fig. 1a). The finding of ref. 6 tends to support the critical-turbulence hypothesis.

The right-side bloom asymmetry is often observed from MODIS and SeaWiFS images^6^,^7^,^8^,^9^,^10^,^11^,^12^,^13^,^14^,^15^, although in some special cases there is no discernible asymmetry [e.g. ref. 7], or even a left-side asymmetry^13^,^15^. On the other hand, blooming may evolve due to the time-dependent nature and forcing of the flow physics and biophysical coupling. This study documents the time-dependency of TC wakes in the western North Pacific using observations from 1998 to 2013. Factors influencing the observed bloom distribution are then explained using model experiments.

## Results

**Figure 1:**
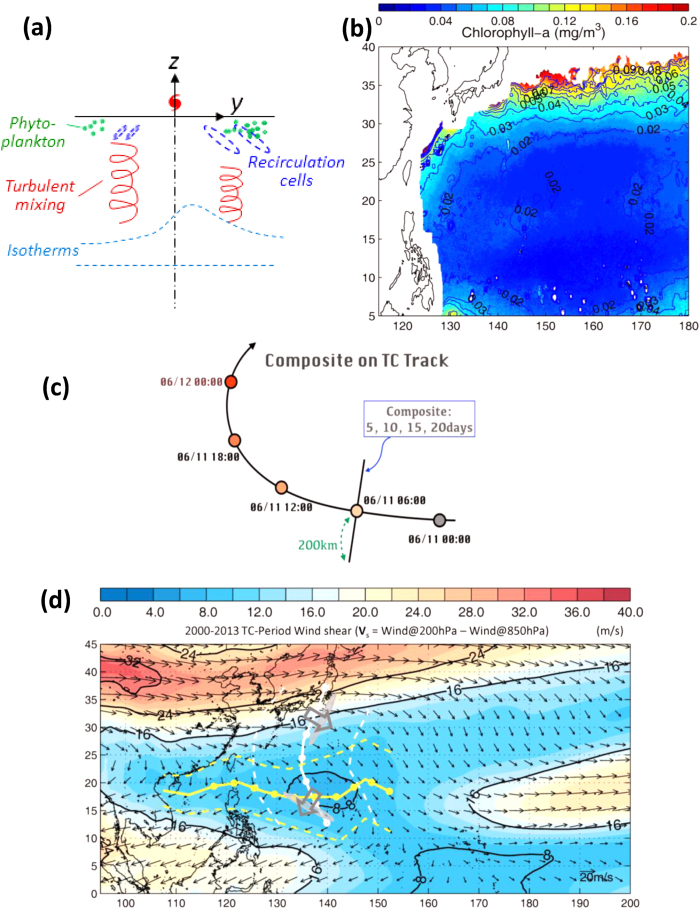
(**a**) A sketch of enhanced right-side (N hemisphere) bloom in TC (symbol, translating into page) wake, due to decreased turbulence by re-stratification of the mixed layer by sub-mesoscale recirculation cells which are stronger on the right [ref. 6]. (**b**) Climatological Chl-a for June to November from 1998 to 2013; contours are the corresponding standard deviations (StD); higher annual StD = 0.037 mg m^−3^ is used to determine the significance of the composite in Fig. 2a,b. See Fig. S1 for rainfall, SST and wind climatologies and StDs. (**c**) A schematic showing how along- and cross-track sections are defined and composites at various *t*_*comp*_ = 5, .. 20 days calculated. (**d**) Climatological environmental vertical wind shear. White and yellow lines are mean typhoon tracks with corresponding ±1StD in dashed lines, for northward and westward translating typhoons, and circles denote mean daily positions. Wide grey arrows are schematic wind shears and down-shear rainfalls are shown in cloud-like shapes. (Maps were plotted using MATLAB Version#R2012a (7.14.0.739) 64-bit (glnxa64).

**Figure 2:**
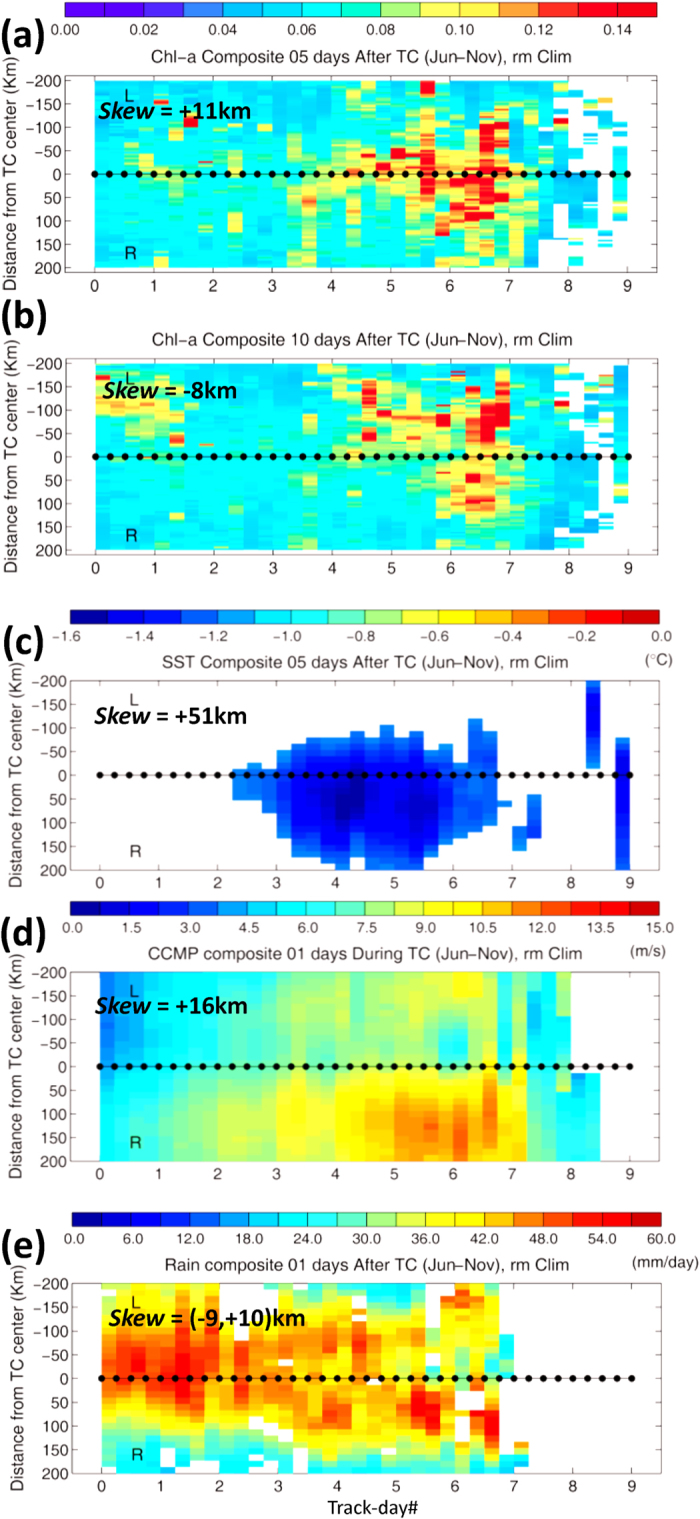
Along- and cross-track composites of Chl-a anomaly for *t*_comp_ = 5 (**a**) & 10 (**b**) days, of (**c**) SST for *t*_comp_ = 5 days, and of wind speed and rainfall (**d,e**) for *t*_comp_ = 1 day. The x-axis is along-track in “Track-day#” and y-axis is cross-track ±200 km to the right (R) and left (L) of the track; track-day0 is when TC first reaches Category 1. Values are shown if anomalies exceed the StDs = 0.037 mg m^−3^, 1.1 °C, 2.8 m s^−1^, and 17 mm day^−1^. “*Skew*” is positive (negative) if composite is rightward- (leftward-) asymmetric; the 2 values for rainfall are for track-day0–4 and track-day4-End.

The method of composite-averaging of variables to the right (+200 km) and left (−200 km) of TC tracks is used (Methods; Supplementary Information Figs S1–S3). At the early phase (*t*_*comp*_ = 5 days), blooming is rightward asymmetric beginning on track-day#3 (Fig. 2a). Here, on average, TCs cross north of 20~25°N and west of 140°E (Fig. 1d) where they reach their maximum intensity (Fig. S4), and depths of upper layer, subsurface chlorophyll maxima, and nutricline depth of maximum Chl-a shallow (Fig. S5), providing favorable conditions for a more vigorous phytoplankton bloom. At the later phase, for *t*_*comp*_ > 5 days, blooming becomes leftward asymmetric (Fig. 2b; Fig. S6). By contrast, SST (Fig. 2c) shows the well-recognized pattern of right-side cooling^5^ irrespective of *t*_*comp*_. The contrast between the time-dependent behaviors of blooming and SST cooling suggests two very different processes.

### Time-dependent bloom asymmetry

Cooling on the right side is produced as more intense mixing due to inertial-current resonance and stronger wind brings cooler subsurface waters to the surface^5^,^6^. Observational evidence of the stronger right-side mixing is a thicker composite of MLD_right_ of about 50 m which exceeds MLD_left_ by about 6 m 2 to 4 days after the TCs (Fig. S9a). The stronger mixing is in part driven by the stronger wind (Fig. 2d), but is predominantly caused by the strong inertial currents due to resonance on the right side^5^,^6^,^21^,^22^,^23^ (Fig. S9b).

At the early phase after the TC-passage (Fig. 2a), while phytoplankton can be enhanced on the right side by the availability of more nutrients entrained from subsurface by stronger mixing (Fig. S10), blooming is predominantly caused by a preferential decrease in turbulence due to a more rapid re-stratification of the mixed layer by sub-mesoscale recirculation cells on the right side^6^ (Fig. 1a). At the later phase (Fig. 2b), however, the left-side asymmetric blooming differs from what is commonly believed. As sub-mesoscale recirculating cells spin down, their impact on re-stratification of the mixed layer weakens, and right-side blooming becomes indistinguishable from the left side. The left-side blooming therefore requires a different explanation. According to the Sverdrup’s critical-depth hypothesis, one could argue that the thinner MLD_left_ may favor enhanced bloom on the left side. However, both MLD_left_ (~45 m) and MLD_right_ (~50 m) are *shallower* than the climatological critical depth *h*_*crit*_ > 100 m [ref. 24]. The difference in MLD left and right of the track is unlikely to cause any preferred blooming.

We attribute the late-phase left-side blooming to rainfall (Fig. 2e). This shows left-side heavier precipitation from track-day 4~5, then becoming right-side heavier from track-day 5~7. The tendency for left-side heavier rainfall can be understood by examining the orientation of TC tracks with respect to the large-scale environmental wind shear (**V**_*s*_ [ref. 25]; Fig. 1d). The **V**_*s*_ is clockwise over the TC region in the western North Pacific, while TC tracks are generally westward and/or north and northeastward. As updraft and rainfall in a TC tend to be generally down-shear^26^,^27^,^28^,^29^, rainfall would then be predominantly on the left side along the westward track and the beginning portion of the northward track, and is predominantly on the right side further north after the TC re-curves along the northward track. The rainfall composites in Fig. 2e are generally consistent with this dependence of the rainfall location on wind shear^25^.

### Observed left-side freshening from ARGO

**Figure 3:**
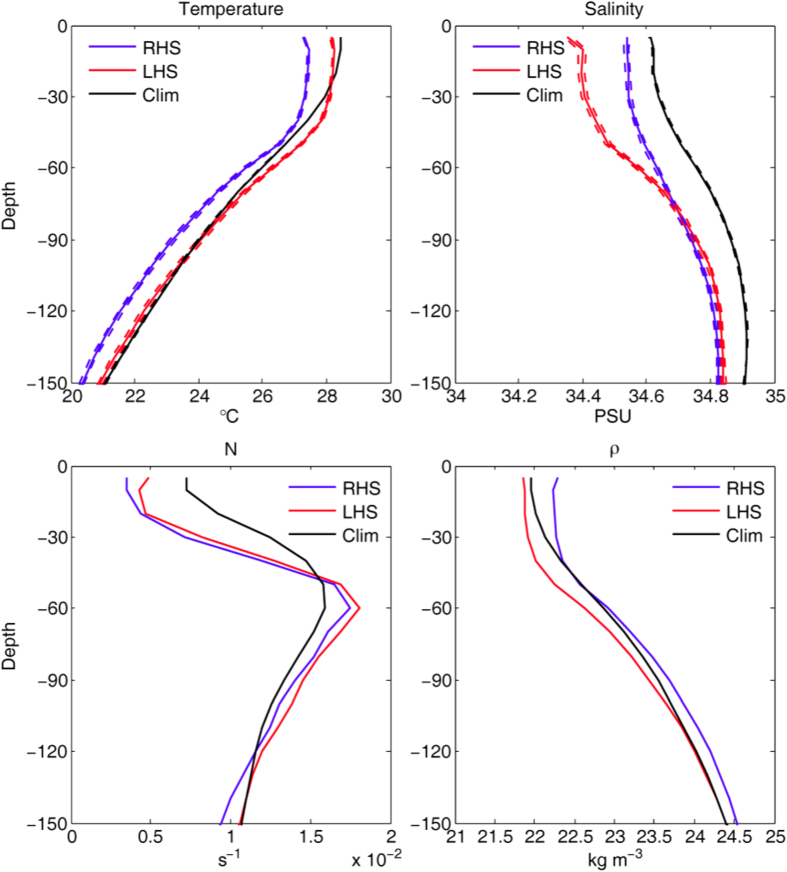
Profiles of the potential temperature (T) and salinity (S), as well as the resulting buoyancy frequency (N) and potential density (ρ = “sigma-theta”), composited from ARGOs within 5 days on the rhs (+200 km, blue lines) and lhs (−200 km, red lines) of all TC tracks. Pre-TC profiles (denoted as “Clim,” black lines) are also composited during 5 days before each TC, taking into account of TC-overlapping (there were only 5).

Rainfall should lead to freshened sea water near the surface. To examine this, we composited ARGO-derived salinity (S) and potential temperature (T) within +200 km on the rhs and −200 km on the lhs, and within 5 days of all TC tracks (Fig. 3). Pre-TC profiles are also composited during 5 days before each TC, taking into account of a very small percentage (<1%) of concurrent overlapping of TC-tracks. The corresponding profiles of potential density ρ and buoyancy frequency (N) are then calculated. The T-profile shows cooling on both sides, but more cooling on the rhs than lhs approximately uniformly in the upper 150 m, consistent with the strong entrainment and upwelling of cooler subsurface waters under the TCs, as well as stronger mixing^5^ and sub-mesoscale recirculation cells^6^ on the rhs. The S-profile shows freshening on both sides of the TCs, but the decrease in surface S with respect to *S*_*clim*_ on the lhs is 3 times more than the corresponding decrease on the rhs: *S*_*lhs*_−*S*_*clim*_ ≈ −0.3 psu, but *S*_*rhs*_ − *S*_*clim*_ ≈ −0.1 psu. We can exclude the likelihood that the freshened *S* is due to entrainment of lower salinity waters from below the depth of the salinity maximum: *S*_*max*_ ≈ 34.9 *psu* at 21 °C (ref. 30; Fig. 3), since this depth is at *z* ≈ −150 m in the tropical waters of the western North Pacific and west Philippines Sea, which is deeper than the MLD^31^ (Fig. S9). We can also exclude the likelihood of significant freshening from riverine waters, since TC tracks within 200 km of the coast are excluded from the analysis. Therefore, the observed surface freshening in the ARGO-composite under the TCs is most likely rainfall-induced. The differenced salinity between the lhs and rhs: *S*_*lhs*_ − *S*_*rhs*_ ≈ −0.2 psu is concentrated in the surface 60 m, i.e. more freshening on the lhs, consistent with the observed rainfall composites that show left-side heavier rainfall (Fig. 2e; Fig. S6). The ρ_*lhs*_ − ρ_*rhs*_ ≈ −0.4 kg m^−3^ is nearly equally contributed by lhs warmer T and freshened S, and N_*lhs*_ − N_*rhs*_ ≈ +10^−3^ s^−1^.

After a storm’s passage, the presence of freshened water near the surface due to rainfall therefore leads to stronger stratification that is preferentially on the left side. Stronger stratification leads to decreased turbulence^32^. As the ocean’s surface layer has been filled with plentiful nutrients entrained from subsurface during the storm, they are preferentially trapped by the stronger surface halocline, favoring phytoplankton blooming on the left side. During the early phase (Fig. 2a), blooming is dominated by the re-stratification effects of sub-mesoscale recirculation cells forced by the powerful near-inertial internal waves^6^, but rainfall influence becomes apparent (Fig. 2b) as the cells weaken at the later phase.

The observational composites (Fig. 2a,b) may include blooming variability due to other processes, e.g. eddies^14^,^33^,^34^, which are ubiquitous in the study region^35^. It is inconceivable however that in general eddies will position themselves preferentially on either side of a TC track to yield a robust composite signal. Most likely, TCs randomly crisscross eddies, and their composite response would tend to cancel given a sufficiently large sample.

### Modeling

**Figure 4:**
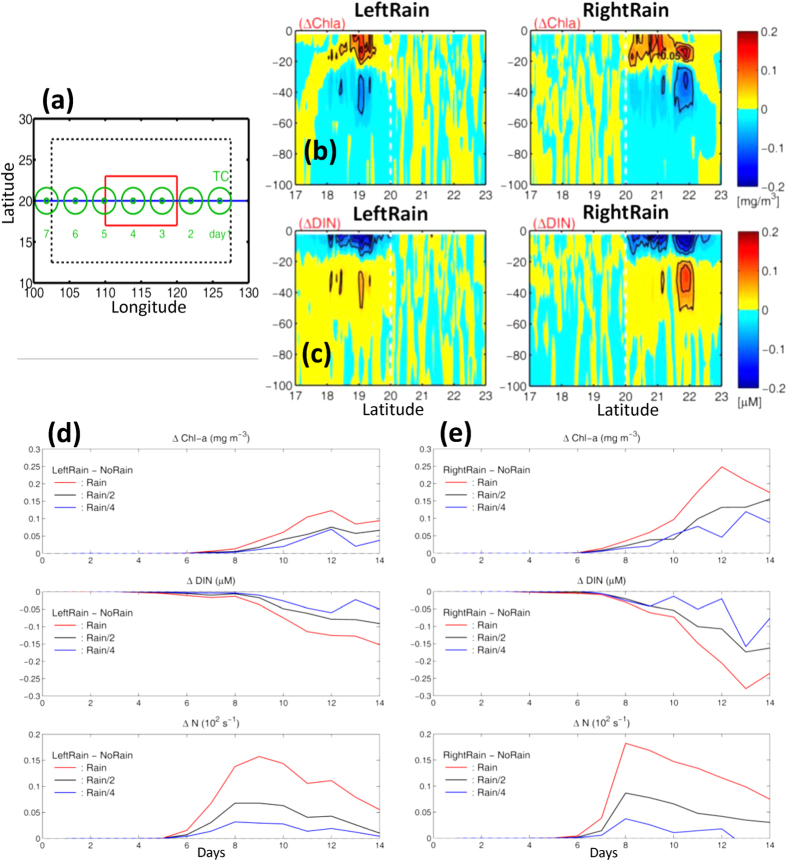
(**a**) Model of a TC translating westward at constant *U* = 5.5 m s^−1^ along 20°N with constant f = 5 × 10^−5^ s^−1^ (blue track with daily green circles) over an idealized 3000 km × 2000 km ocean basin of constant depth 2000 m; grid sizes Δ*x* = Δ*y* = 2.5 km and Δ*z* = 5 m in the top 100 m; longitude 100–130°E are for reference only. Dotted line shows “sponge layers” to minimize reflections into the red center domain. Ocean is initially at rest with T, Chl-a etc function of z only from climatology. Model results at day 10 are plotted as differences of LeftRain minus NoRain and RightRain minus NoRain experiments for (**b**) Chl-a and (**c**) DIN. TC center is at the vertical dashed line, view is in the TC direction, and variables are zonally averaged from 110°–120°E in the red center domain. (**d**,**e**) LeftRain & RightRain minus NoRain differenced Chl-a, DIN & N averaged from *z* = 0 to *z* = −25 m on left (**d**) and right sides (**e**) of the red domain; abscissa is time (days). Note that as the model TC passes the red domain from day 3~5, then e.g. “day 10” means “6 days after” the TC, etc. Colored lines are for different rain rates = 20 (Rain), 10 (Rain/2) and 5 (Rain/4) mm day^−1^ when the TC passes through the red center domain.

Rainfall-enhanced blooming is quantified using the Huang and Oey’s^6^ model for simulating biophysical response in the wake of a TC (Fig. 4a; Supplementary Information). The RightRain and LeftRain experiments specify rain to the right and left respectively of the cyclone, following the storm as it passes through the red center domain (Methods). Rain minus NoRain differences in Chl-a (Fig. 4b) show that rain increases the near-surface blooming on the rain side, while the dissolved inorganic nitrogen (DIN) *decreases* (Fig. 4c). The halocline traps nutrients which are continually being depleted as phytoplankton blooms, and at the same time dampens mixing that would otherwise have entrained more nutrients to the surface. Effects of rain are confined to the side where rain is specified: within the model integration period (20 days), the double fronts formed in the wake have not collapsed^6^, and there is little exchange across the center. The dependence of rainfall-enhanced blooming on stratification is illustrated in Fig. 4d,e for 3 different rainrates. The model produces increased buoyancy-frequency responses due to the rainfall, ΔN, which ranges from 3 × 10^−4^ s^−1^ (for “Rain/4”) to 1.8 × 10^−3^ s^−1^ (for “Rain”), consistent with the observed response of N_*lhs*_ − N_*rhs*_ ≈ +10^−3^ s^−1^ from the ARGO composite (Fig. 3). The enhanced blooming (ΔChl-a) appears 4~6 days after the typhoon has passed (at model day 8~10), consistent with the late-phase rainfall-enhanced blooming which in the case of the observed Chl-a appears approximately 5 days after the TC-passage on the left side (Fig. 2b). Rainfall- enhanced blooming lags increased buoyancy frequency (ΔN) by 2~4 days, at the expense of DIN, which decreases in phase with the increased Chl-a. These behaviors are generally similar for the RightRain experiment, but they are stronger for the same rainrate (Fig. 4e) because of contributions from the sub-mesoscale recirculation cells^6^. Larger rainrates also produce stronger responses, and the changes are approximately linear (i.e. doubling the rainrate doubles also ΔChl-a, |ΔDIN| and ΔN) except for the experiments with the weakest rainrate, probably because the stratification effects of sub-mesoscale cells can then still dominate the solution.

## Discussions

In conclusion, 16-year composite of satellite data in typhoon wakes shows a right-side phytoplankton bloom at the early phase, 1~5 days after the storm, but an unexpected enhanced blooming on the left side at a later phase, which we explain is due to the ocean’s stratification by enhanced rainfall on the left side of the storm, and which has thus far been overlooked in the literature. The enhanced stratification preferentially traps nutrients near the surface, leading to the enhanced blooming. Our results suggest the critical importance of the large-scale environmental wind shear which affects the rainfall pattern; in turn, the latter controls the bloom location and intensity, contributing to their spatial coherency (Fig. S2b,c). We applied the same composite analysis to the western North Atlantic, where the large-scale wind shear is nearly uniformly westerly over the northward re-curving hurricane tracks (Fig. S11a), yielding a down-shear rainfall pattern which is predominantly on the right of the tracks (Fig. S11d). In contrast to the western North Pacific, blooming is now right-side asymmetric both in early and late phases (Fig. S11b,c).

Large-scale increased stratification in the tropics and subtropics due to climate warming has been blamed for the long-term decline in phytoplankton^3^,^4^,^36^. Despite its short life, TC-induced blooming, because of its great intensity, may increasingly contribute to the long-term variability of the ocean’s net primary production^16^. As the ocean warms, stronger TCs may be anticipated in the future^37^,^38^,^39^,^40^, with stronger mixing and heavier precipitation which promote stronger phytoplankton response in their wakes. The present work has shed some light to understanding the biophysical interplay between blooming and various environmental factors, but more research is needed to assess the impact on climate, and *vice versa*.

### Data

Surface Chl-a data is from the Ocean Color Climate Change Initiative project on 4 km × 4 km grid (http://www.esa-oceancolour-cci.org/). The wind velocity is from the Cross-Calibrated Multi-Platform (CCMP^41^), sea surface temperature (SST) from the Group for High Resolution Sea Surface Temperature (GHRSST^42^), and precipitation data from the Tropical Rainfall Measuring Mission (TRMM, 3B42 v7), all on 1/4° × 1/4° grids. These data are composited following the tracks of typhoons obtained from the International Best Track Archive for Climate Stewardship data [IBTrACS^43^]. The CCMP wind field and IBTrACS track data are 6-hourly; other data are daily.

Other data used include: ARGO temperature profiles from 1999 to 2013 to calculate a measure of the mixed layer depths (MLD) as the depth where the temperature is 0.2 °C less than the surface^44^, before and after the passages of TCs; the National Centers for Environmental Prediction (NCEP) global Forecast system (GFS) final (FNL) 1° × 1° gridded analysis datasets from 2001–2013 to composite the vertical wind shear (200 mb–850 mb); and satellite-tracked surface drifters from the Global Drifter Program dataset (ftp.aoml.noaa.gov) from 1998 to 2013 to estimate the TC-induced inertial currents.

## Methods

Arithmetic averaging of a large sample of events (composite analysis), used in many subfields of atmospheric, oceanic and climate sciences to identify dominant patterns^10^,^16^,^45^,^46^,^47^,^48^,^49^,^50^, is applied to Chl-a and other variables over the TC tracks. The Chl-a is composited from June through November of 1998 to 2013, in the western North Pacific at least 200 km from the coast and south of ~35°N where the Chl-a is generally low^3^ (Fig. 1b; Supplementary Information). Blooming is considered significant only if it exceeds Climatology + 1StD, where StD = 0.037 mg m^−3^ is the domain-averaged seasonal standard deviation (Fig. S1), and Climatology is monthly climatology interpolated to the location and time of the TC. This threshold value is comparable to (less stringent than) that used in refs 8 & 10 (refs 16 and 33) to distinguish between pre- and aft-TC Chl-a. To test sensitivity, the analysis is repeated using a threshold of 2 × StD (which are now more stringent than the values used in refs 16 and 33). The higher threshold has the effect of placing more weights on fewer typhoons with apparently stronger blooming *or* more complete data; otherwise our finding of the late-phase, leftward shifting in bloom is unchanged (Fig. 2a,b and Fig. S2g,h). For rainfall rate, SST and wind speeds, their seasonal StDs are used to define the significance of the corresponding TC-induced signals (Fig. S1).

We map all the data onto a uniform along- and cross-track (±200 km, right and left) grid based on each TC track (Fig. 1c; Fig. S2). In Fig. 2, we show only TC tracks after the storms have reached typhoon strength Category-1 with wind speed >33 m/s [ref. 51]; but tracks at all stages of the storms including the “tropical storm” status (>17 m/s but less than 33 m/s) have also been analyzed, indicating weaker blooming for TC-strength weaker than Category-1 (Fig. S2d,e). A total of 141 TC tracks (Fig. 1d; Fig. S3) were analyzed. In the composite, the along-track distance is displayed as “track-day#” with track-day0 at the point when the TC first reaches the Category1 or tropical storm status (Fig. 2, or Fig. S2d,e), and with equal ¼ day interval for all TCs. Using an averaged *U* = 5.5 m s^−1^ for all 141 TCs (Fig. S3), one track-day ≈ 475 km. At each grid location, we composite the anomalies for *t*_comp_ days after the TC has passed the grid point: the composite is “Lagrangian” following the TC, and the composite period after the TC-passage is the same for all points along the track (examples in Fig. S2f). This differs from the traditional method of composite for anecdotal events^6^,^7^,^8^,^9^,^11^,^12^,^13^,^14^,^15^, which fixes the calendar period instead, yielding different duration times after TC-passage for different locations. We repeat the procedure for each TC and then average over all of them. The anomalies are defined as instantaneous values at the time of the TC-passage minus the climatologies.

The right or left-side asymmetry is measured by a skewness parameter *Skew*(*v*) = ∫(*yv*)*dy*/∫*vdy* in *km*, where *v* = ∑(Chl-a) for e.g. the Chl-a composite, ∑ = along-track sum, and the integral is taken across-track from *y* = −200 *km* to +200 *km*; thus *Skew* is the “*y-*center of mass” of the composite. Positive (negative) skewness indicates that the pattern is right-side (left-side) asymmetric.

The climatological wind shear (Fig. 1d) is calculated by first averaging the magnitude |**V**_s_| and direction of the daily shear for the duration of each TC *excluding* ±200 km of the track^25^. The averaging of |**V**_s_| ensures that magnitude of large-scale shear for each TC is preserved. We then composite these quantities for all the TCs.

The model is modified from that detailed in ref. 6 (see Supporting Information) to include rainfall, as follows. The rainfall is applied mimicking the rainfall in a real TC: moving under the translating, circular model TC from the time it enters the analysis center (red) domain, to the time when it exits (Fig. 4a), and it is spread within 200 km radius (r) either to the left (i.e. left half-circle: LeftRain) or right (right half-circle: RightRain) of the track as a Heaviside-function: RainRate × (H_v_(r) − H_v_(r − 200 km)), where H_v_(α) = 0 (1) for α ≤ (>) 0. The specified rain rate is typical of the values found under a TC^29^,^52^. To test sensitivity, other experiments with reduced Rain/2 and Rain/4 are also reported in the text.

## Additional Information

**How to cite this article**: Lin, Y.-C. and Oey, L.-Y. Rainfall-enhanced blooming in typhoon wakes. *Sci. Rep.* **6**, 31310; doi: 10.1038/srep31310 (2016).

## Supplementary Material

Supplementary Information
