# Supplementary material for: Rainfall-enhanced blooming in typhoon wakes
Source: Sci Rep. 2016 Aug 22;6:31310. doi: 10.1038/srep31310 (PMC4992858; doi:10.1038/srep31310)
Supplement: Supplementary Information [file srep31310-s1.pdf]

Supplementary Information for

Rainfall-enhanced blooming in typhoon wakes

Y.-C. Lin and L.-Y. Oey*
National Central University; *Corresponding Author: lyooey@gmail.com

Contents of this file

Text S1. Climatology and standard deviations
Text S2. Examples of composite maps
Text S3. Typhoon tracks
Text S4. Location of TC lifetime maximum intensity
Text S5. Z_{22} and subsurface chlorophyll maximum
Text S6. Rainfall composites
Text S7. ARGO data and MLD climatology
Text S8. One-dimensional (z-only) biophysical model
Text S9. Huang and Oey [ref.6] biophysical ocean model

Figures S1 to S11

Introduction

This Supplementary Information file includes supporting text and figures which describe the observations in more details. It also describes in details the biophysical model used in the main text.

Text S1. Climatology and standard deviations

The June-November climatological means of the above fields and their standard deviations (StD) are shown in Fig.S1. The Chl-a is generally low in the oligotrophic region of interest south of approximately the 35°N in the western North Pacific. The area-averaged (mean, StD) are ≈ (0.065, 0.028) mg m^{-3}, but for the purpose of determining the significance of the Chl-a composite, the larger seasonal (i.e. annual) StD = 0.037 mg m^{-3} is used. The seasonal StD is still much lower than the Chl-a blooming observed in the wakes of TCs, which are typically 5~20 times stronger[6-15]. A more stringent value of 2×StD is also tested to show the insensitivity of the results, as described in text. To avoid regions of high Chl-a unrelated to TCs, we use only those TC tracks which are over the region where the seasonal StD of Chl-a is less than 0.1 mg m^{-3} and at least 200 km from the coast (where satellite values are less reliable and anthropogenic inputs are high; Fig.S1a). This "mask" based on Chl-a is used for analyses done on all the other field variables. The climatology SST (Fig.S1b) decreases with latitude with an area mean of 27.7 ^{o}C and a seasonal StD = 1.1 ^{o}C. The climatology wind speed shows a zonal pattern, with an area-mean of 8 m/s and seasonal StD of 2.8m/s (Fig.S1c). The area-mean precipitation is 5.4 mm day^{-1} and StD is 13 mm day^{-1} (Fig.S1c). We will see that rainfall is an important parameter for blooming. We therefore use a more stringent StD value of

17 mm day^{-1}, which is the area mean of the large StD values from 5~15°N, to determine the significance of TC-induced precipitation.

Text S2. Chl-a and rainfall composites

Anecdotal examples of Chl-a and rainfall composites are shown in Fig.S2a-f to further clarify the methodologies used in the main text. The correlations between Chl-a and rainfall, for both the western North Pacific and Atlantic, are shown in Fig.2b,c. Total Chl-a composite using a threshold of 2×StD is shown in Fig.S2g,h.

Fig.S2a (middle and bottom panels): these show examples the results of the TC-following ("Lagrangian") composite t_{comp} = 10 days for two TCs: northward re-curving TC (middle) and westward TC (bottom).

Fig.S2b,c: these show two examples of t_{comp} = 10day Chl-a composites (top) and the corresponding t_{comp} = 1day rainfall composites (middle). From composite maps like these, one pair of maps for each TC, we calculate the correlation between Chl-a and rainfall (bottom). Despite the noisy patterns and large amount of missing pixels for both variables, nearly all of the correlation values are positive and most are significant at the 95% confidence level. The correlation ranges from near-zero (insignificant) to 0.65 with a mean for all TCs from 1998 to 2013 of about 0.3, significant at the 95% confidence level, both for the western North Pacific typhoons and for the Atlantic hurricanes. The positive correlations indicate that rainfall and blooming patterns tend to be spatially coherent.

Fig.S2d,e: these show examples of Chl-a on along-track and cross-track transformed maps for 9 TCs, and their total composite (last panel on bottom right). Note the general change in the blooming pattern to become more leftward asymmetric from early phase (Fig.S2d) to late phase (Fig.S2e).

Fig.2f: these show examples of what Chl-a composites on the transformed maps look like when plotted on the geographical maps.

Fig.2g,h: these show the results when a threshold of 2×StD is used (instead of 1×StD) to calculate the total composite of Chl-a anomalies for all TCs from 1998-2013. Comparing to Fig.2a,b in the main text (using 1×StD), the change to a more leftward asymmetric blooming pattern from early phase (Fig.S2g) to late phase (Fig.S2h) can also be seen.

Text S3. Typhoon tracks

Typhoon tracks are grouped into westward- and northward-translating groups in Fig.S3a,b, and TC#s and translation speeds in Fig.S3c,d. The translation speeds of TCs (Fig.S3d) show that the majority of them travel at supercritical speeds $U/C > 1$ [ref.53], faster than the 1st mode internal phase speed of the ocean, $C \approx 3$ m/s in the western North Pacific subtropical region[54]. Eleven (of 141 or ~8%) of them travel below C

Text S4. Location of TC lifetime maximum intensity

The International Best Track Archive for Climate Stewardship (IBTrACS) data[43] is used to compute the locations of TC lifetime maximum intensity in the western North Pacific. They are annual averaged and plotted in Fig.S4, showing that the TCs are most intense when they reach near 20~25°N and west of 140°E. There is also a poleward-shifting trend of the location so that in recent decades TCs tend to reach their maximum intensity at locations which are further northward[47].

Text S5. Z_{22} and subsurface chlorophyll maximum

ARGO data is used to calculate the depth of the 22 ^{o}C isotherm Z_{22} (Fig.S5a). WOA data is used to calculate the depth of the subsurface chlorophyll maximum (Fig.S5b).

Text S6. Rainfall composites

Chlorophyll and rainfall composites for westward (Fig.S6a,b,c) and northward (Fig.S6d,e,f) translating typhoon groups show similar early-phase right-side and late-phase left-side Chl-a asymmetries, as well as left-side rainfall asymmetry as for the corresponding total composites (Fig.2).

Text S7. ARGO data and MLD climatology

A knowledge of mixed layer depth (MLD) is necessary to understand the phytoplankton and SST responses to TCs. The MLD is defined as depth where the temperature is 0.2 ^{o}C less than the surface[44]. We calculate MLD using the ARGO data from 1999-2014. The number of ARGOs and the long-term averaged mixed layer depth (MLD) in the study domain are shown in Fig.S7. In Fig.S7a, the number of ARGOs in 5 ^{o}×5 ^{o} grids is shown as a function of years. Rich-ARGO years are from 2003 to 2012 when there were more than 200 per year in the study region. The long-term averaged MLD (Fig.S7b) in the study region is 34.59 m, and shows generally larger values south of ~20°N than north, except near the Kuroshio extension where the MLD can be large in localized regions.

The seasonal MLD (Fig.S8) shows shallow isotherms in the zonal band from 18~27°N in winter (Fig.S8a) coinciding with the subtropical counter current (STCC) [see Fig.1b of ref.35], sandwiched between a ridge to the north near the Kuroshio, and a ridge to the south from 8~18°N. Both ridges weaken in spring (Fig.S8b). The northern ridge all but disappears in summer~fall, while the southern ridge weakens but the MLD remains deeper than ~50 m (Fig.S8c,d). This thick MLD overlies a warm upper-layer water whose temperature exceeds > 22 ^{o}C through 200 m below the surface in this region (Fig.S5), and explains why the composite SST cooling is insignificant during the first 3 days of the track (Fig.2c) despite very strong winds under the TCs over the same region. North of 18°N, after 3~4 days on the composite track, the MLD thins to less than 20~30 m. Coupled with the shallow Z_{22} to the north, significant SST cooling is produced.

After the passage of a TC, the MLD becomes thicker on the right than left side of the track. We wish to quantify this using the ARGO data. However, the data is insufficient for the track-composite analysis. We therefore composite instead ARGOs which are within ±200km left and right of the storm's track (Fig.S9a). There is almost no difference for the averaged MLD on the left and right sides of the track before TCs (0 day), and the values ≈ 36~38 m are close to the climatological MLD. The difference between right and left sides increases after the passage of the TC, and at day 4, the $MLD_{right} > MLD_{left}$ by about 6 m, indicating stronger mixing on the right side produced by the TCs. Both MLD_{left} and MLD_{right} merge to the same background value of 35~40 m in about 8 days, consistent with the results of Foltz et al [ref.16].

The stronger right-side mixing is in part driven by the stronger wind on the right side (Fig.2d), but it is predominantly driven by strong inertial currents due to resonance[5,6,21-23]. We can quantify this using surface drifter data following the same analysis as for the ARGO data. The inertial current amplitude (i.e. speed) is calculated from the drifters using the method described in refs.55-56. The day0 speed is taken to be the same as the background climatological inertial current speed which is ≈ 0.2 m s^{-1}, and which agrees well with that reported ref.57 for our study region. Generally stronger inertial current is on the right side, reaching a maximum of about 0.5 m s^{-1} around day 2 (Fig.S9b). It is interesting that after an initial rapid decay from day 3~8, the inertial current speed remains strong in the wake of the TC, about 0.33 m s^{-1}. We checked that this was not caused by typhoons which overlapped in time. The reason is unclear, but it may be because very stratified surface layer remains in the wake due to TC rainfall (see text), and inertial currents tend to become amplified in the thin stratified surface layer[58].

Text S8. One-dimensional (z-only) biophysical model

Model of Huang and Oey [ref.6] was run to simulate the effects of 1-D mixing on blooming (Fig.S10). This shows that vertical mixing alone produces only a weak asymmetry: first because mixing brings Chl-a from its subsurface maximum to the surface, and secondly because nutrient is also mixed to the surface and blooming ensues.

Text S9. Huang and Oey [ref.6] biophysical ocean model

The model is described in detail in ref.6. For the present application, rain is specified as described below. The physical model is the parallelized version of the time-dependent and three-dimensional Princeton Ocean Model[59,60], and the biological model is based on the NPZ (nitrogen, phosphate and zooplankton) model of Liu et al. [refs.61-63]. Our goal is to study processes, and the same idealized model domain and setting of Huang and Oey [ref.6] are used (Fig.3a). An f-plane centered at 20°N ($f = 5\times10^{-5}$ s^{-1}) is used in a rectangular closed ocean of constant depth H = 2000 m with zonal and latitudinal dimensions 3000 km × 2000 km. The latitude is therefore shifted poleward by 5° from 15°N used by Huang and Oey who used the model for South China Sea, and initial profiles of temperature, nitrogen and chlorophyll are also accordingly modified for the western North Pacific (see below). The model has 61 vertical levels with fine grid sizes Δz = 5 m in the top 100 m, and horizontal grid size $\Delta x = \Delta y$ = 2.5 km, where *x, y* and *z* are

zonal, meridional and vertical axes. A symmetrical vortex is used to model the typhoon[64], specified with a maximum wind speed = 60 m s^{-1} at radius 50 km from the center, quite typical of the radius of TC eye[29], and made to translate at a constant speed U = 5.5 m s^{-1} from east to west along the middle of the model ocean basin. The speed of 5.5 m s^{-1} equals to the average translating speed of all the observed typhoons used in the composite analyses, i.e. it is the (weighted) mean of the translating speeds of westward and northward TCs in Fig.S2 or Fig.1d. The ocean is initially at rest and stratified, with potential temperature a function of *z* only chosen from the WOA climatology averaged from June-November and over the region of the western North Pacific shown in Fig.1a. The chlorophyll and nitrogen profiles are likewise functions of *z* only, also chosen from the WOA dataset. The integration is carried out for 20 days, and the results below are analyzed in the "analysis center domain" (red rectangle) shown in Fig.3a, away from side boundaries, and through which the model TC enters at day 3 and exits at day 5. NoRain, RightRain, LeftRain and AllRain experiments were carried out, although only the first 3 are used in the text. The AllRain experiment was used to confirm that there was indeed little exchange between left and right. The basic rain rate (denoted as "Rain" in text Fig.3d,e) is 20 mm hr^{-1}. The rainfall is applied mimicking the rainfall in a real TC: moving under the translating, circular model TC from the time it enters the analysis center (red) domain, to the time when it exits (Fig.4a), and it is spread within 200 km radius (r) either to the left (i.e. left half-circle: LeftRain) or right (right half-circle: RightRain) of the track as a Heaviside-function: RainRate × (H_v(r)-H_v(r-200km)), where $H_v(\alpha) = 0$ (1) for $\alpha \leq (>) 0$. The specified rain rate is typical of the values found under a TC[29,52]. To test sensitivity, other experiments with reduced Rain/2 and Rain/4 are also reported in the text.

References

53. Oey, L.-Y., Ezer, T., Wang, D.-P., Yin, X.-Q. & Fan, S.-J. Hurricane-induced motions and interaction with ocean currents. *Cont. Shelf Res.* **27**, 1249-1263, doi:10.1016/j.csr.2007. 01.008 (2007).
54. Chelton, D.B., deSzoeke, R. A., Schlax, M. G., Naggar, K. & Siwertz, N. Geographical variability of the first-baroclinic Rossby radius of deformation. *J. Phys. Oceanogr*. **28**, 433–460 (1998).
55. Fan, S., Oey, L.-Y. & Hamilton, P. Assimilation of drifter and satellite data in a model of the Northeastern Gulf of Mexico. *Cont. Shelf Res*. **24(9)**, 1001-1013 (2004).
56. Wang, D.-P. & Oey, L.-Y. Hindcast of Waves and Currents in Hurricane Katrina. *Bull Amer. Meteor. Soc.* **89**, 487-495 (2008).
57. Chaigneau, A., Pizarro, O. & Rojas, W. Global climatology of near-inertial current characteristics from Lagrangian observations. *Geophys. Res. Lett.* **35**, L13603, doi:10.1029/2008GL034060 (2008).
58. Gill, A. E. Atmosphere-Ocean Dynamics. Academic Press, New York. 662 pp (1982).
59. Oey, L. et al. ATOP – The Advanced Taiwan Ocean Prediction System based on the mpiPOM. Part 1: model descriptions, analyses and results. *Terr. Atmos. Ocean. Sci.* **24**, 137-158, doi: 10.3319/TAO.2012.09.12.01(Oc) (2013).

60. Oey, L.-Y. et al. Cross flows in the Taiwan Strait in winter. *J. Phys. Oceanogr.* **44**, 801-817 (2014).
61. Liu, K. K. et al. Monsoon-forced chlorophyll distribution and primary production in the South China Sea: observations and a numerical study. *Deep-Sea Res. I.* **49(8)**, 1387-1412 (2002).
62. Liu, K.-K. et al. The significance of phytoplankton photo-adaptation and benthic-pelagic coupling to primary production in the South China Sea: Observations and numerical investigations. *Deep-Sea Res. II*. **54**, 1546–1574 (2007).
63. Liu, K. K. et al. Inter-annual variation of chlorophyll in northern South China Sea observed at the SEATS Station and its asymmetric responses to climate oscillation. *BiogeoSci*. **10(11)**, 7449-7462 (2013).
64. Holland, G. J. An analytic model of the wind and pressure profiles in hurricanes. *Mon Wea Rev*. **108**, 1212-1218 (1980).

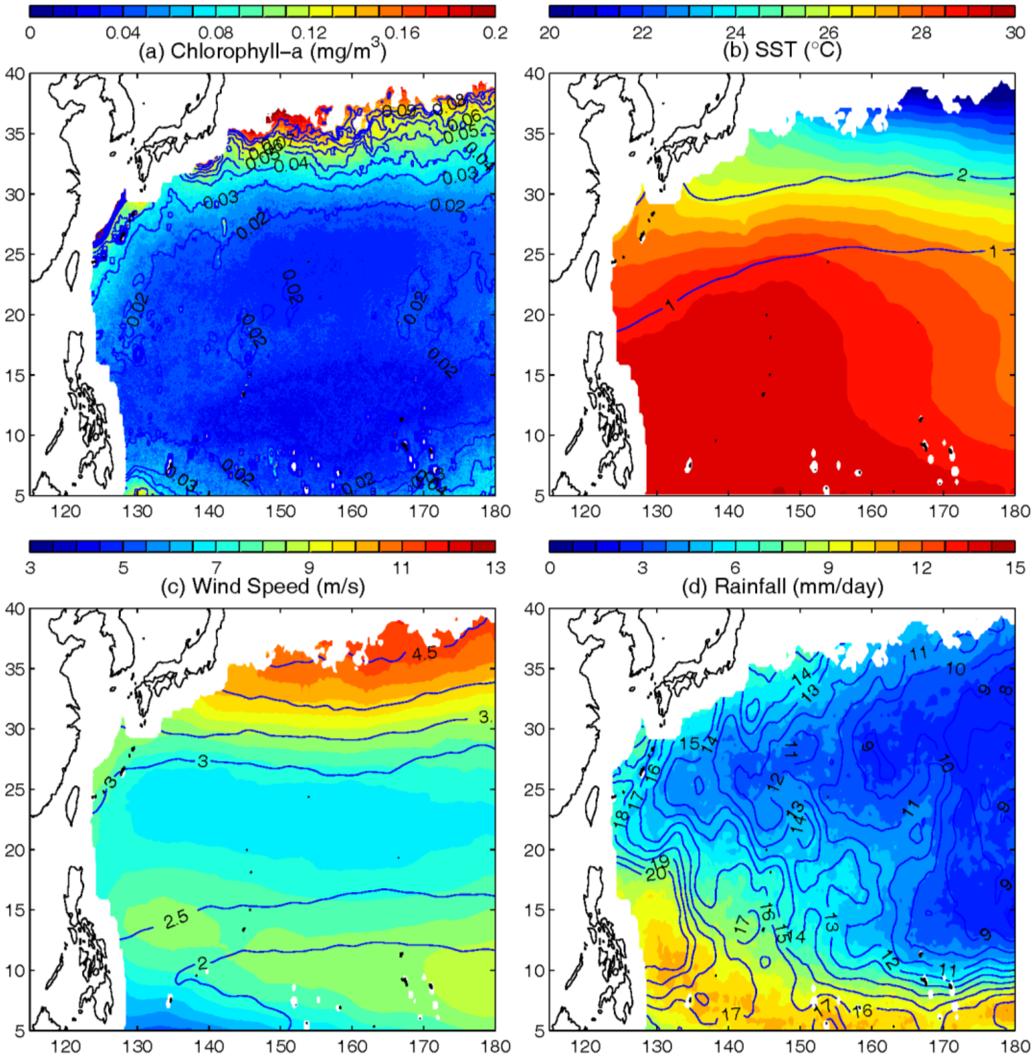

Fig.S1: Climatological Chl-a (a), SST (b), wind speed (c) and rainfall (d) calculated for June to November from 1998 to 2013. Contours indicate the standard deviations (StDs). The *seasonal* (i.e. annual over the year, not just for Jun-Nov) StDs for the 4 fields averaged over the region are: 0.037 mg m^{-3}, 1.1 ^{o}C, 2.8 m s^{-1}, and 13 mm day^{-1}. (Maps were plotted using MATLAB Version#R2012a (7.14.0.739) 64-bit (glnxa64) http://www.mathworks.com/support/compilers/R2012a/glnxa64.html).

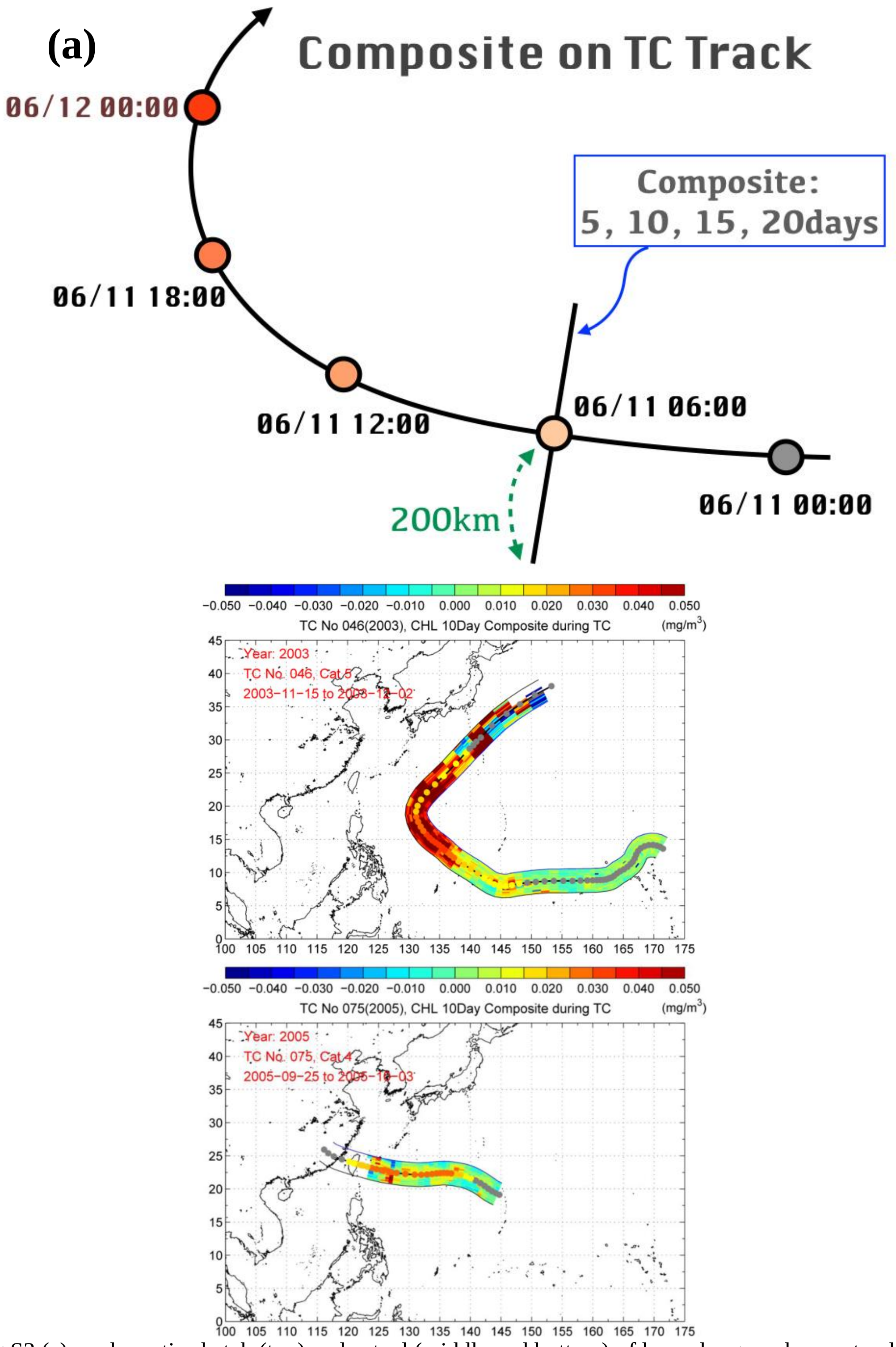

Fig.S2 (a) a schematic sketch (top) and actual (middle and bottom) of how along- and cross-track sections are defined and composites at various t_{comp} = 5, 10, 15 and 20 days calculated. The middle and bottom panels show examples of Chl-a composite for t_{comp} = 10 days and 2 different typhoon tracks (TC periods are indicated). Dots on each track are 6-hourly and grey dots indicate locations where the storm is still a tropical storm with wind speeds greater than 17 m s^{-1} but below 33 m s^{-1}. (Maps were plotted using MATLAB Version#R2012a (7.14.0.739) 64-bit (glnxa64) http://www.mathworks.com/support/compilers/R2012a/glnxa64.html).

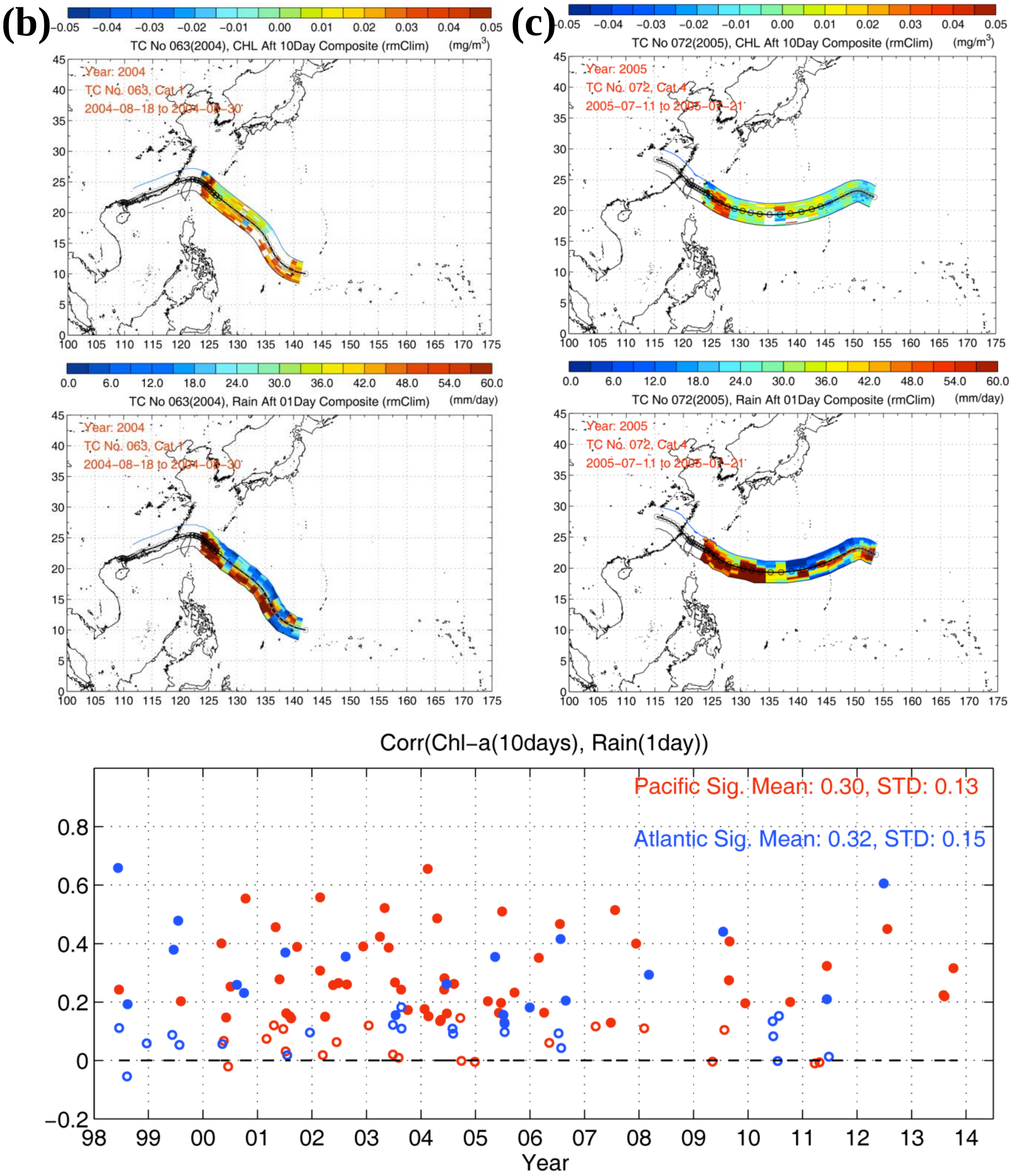

Fig.S2b,c Two examples (b & c) of typhoon-induced Chl-a (top) and rainfall (middle) composites, showing left-side blooming and rainfall preferences. The t_{comp} = 10 days for Chl-a and = 1 day for rainfall. TC periods are indicated. The time-series plot in the bottom panel shows correlation between Chl-a (t_{comp} = 10 days) and rainfall (t_{comp} = 1 day) for TCs with more than 25% (approximately 300~500 points) spatially overlapping (i.e. simultaneous) pixels along the TC track, from 1998 to 2013. Filled (open) symbols show values significant (insignificant) at the 95% confidence level, and red (blue) is for the western North Pacific (Atlantic) TCs, and their mean correlations and StD are also shown. (Maps were plotted using MATLAB Version#R2012a (7.14.0.739) 64-bit (glnxa64) http://www.mathworks.com/support/compilers/R2012a/glnxa64.html).

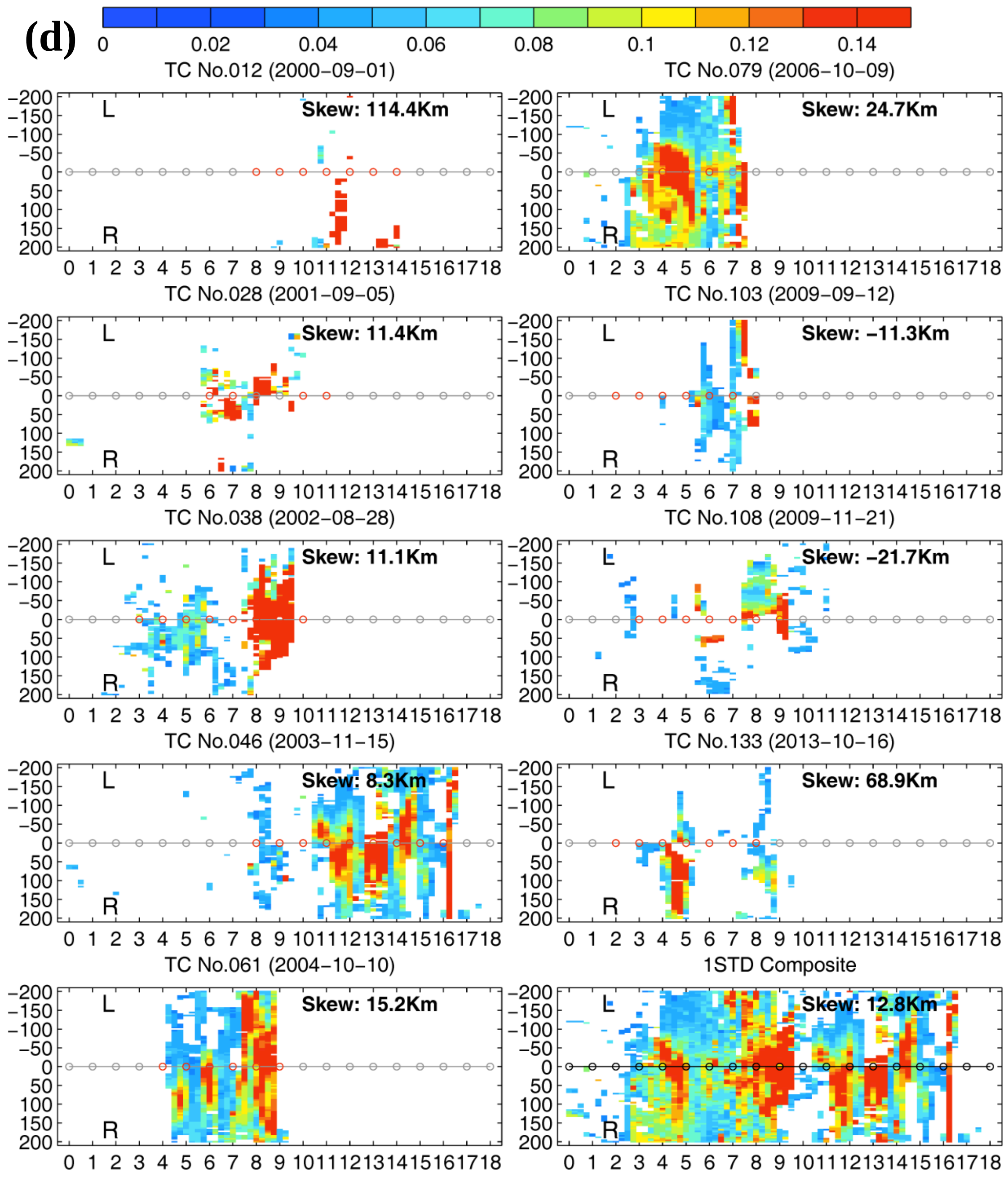

Fig.S2d Examples of 9 typhoon-induced Chl-a (anomaly in mg m^{-3}) blooming (left 5 panels and 1st 4 panels on right) composited for t_{comp} = 5 days (i.e. early phase) and mapped into uniform along-track (abscissa “x” in “track-days”; track-day0 is when TC first reaches tropical storm status) and cross-track (ordinate “y” in “km”) domain. Grey (red) circles along the track at y = 0 indicate tropical storm (Category1 and above) status. Note that blooming occurs mainly when TC strength reaches Category 1 (or above). The Skew is shown on each panel. Bottom right panel shows the composite of all 9 events, indicating Skew = +12 km or a rightward bias for these 9 events in the early phase.

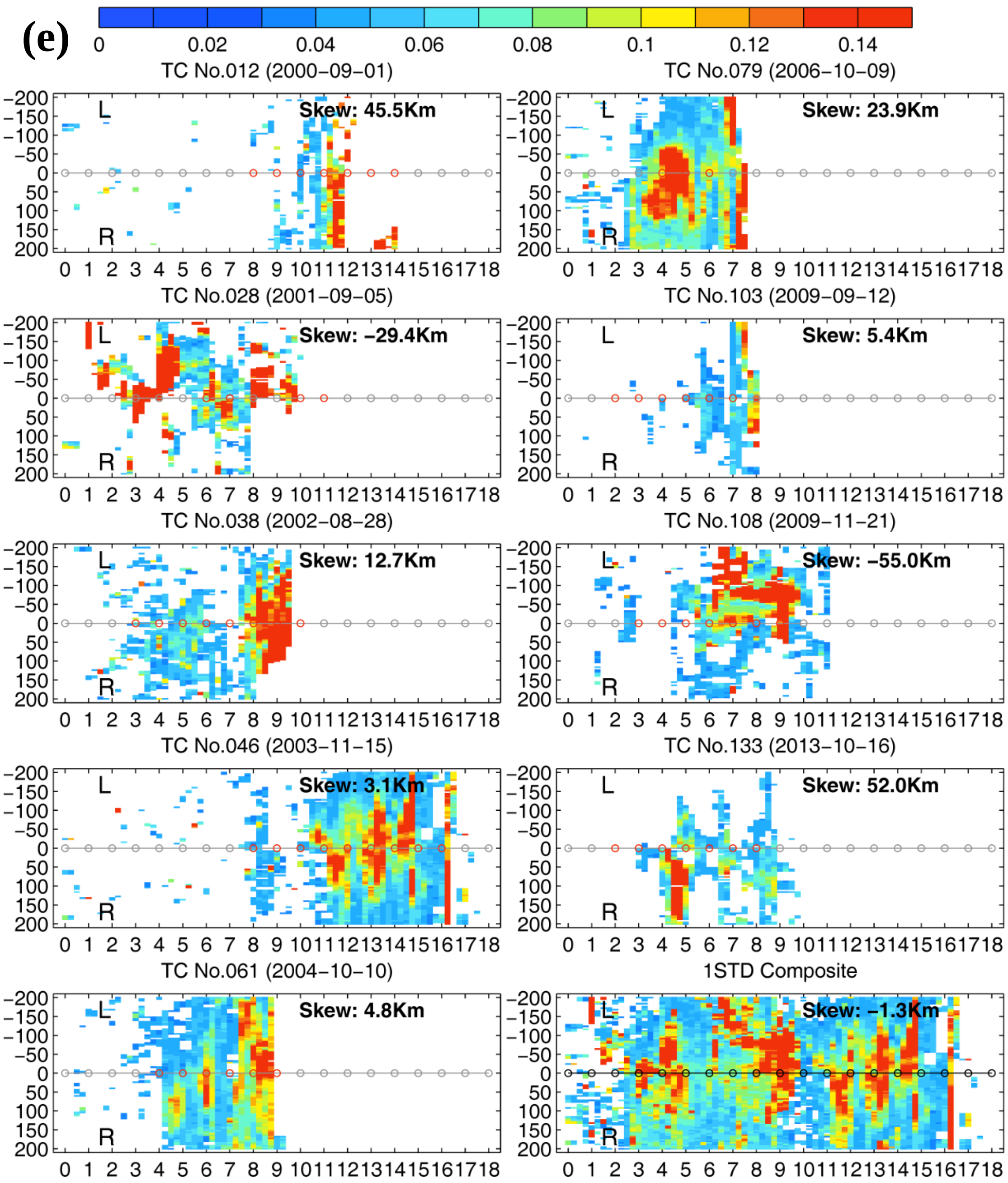

Fig.S2e Same as Fig.S2d for t_{comp} = 10 days (i.e. late phase). Bottom right panel shows the composite of all 9 events, indicating for these 9 events a weak Skew = -1 km; comparing it with Fig.S2d, the composite shows the tendency for a left-side shift in blooming at the late phase. These examples also indicate that slow-moving storms (TC#28 and 108; see Fig.S3d) can produce large blooms.

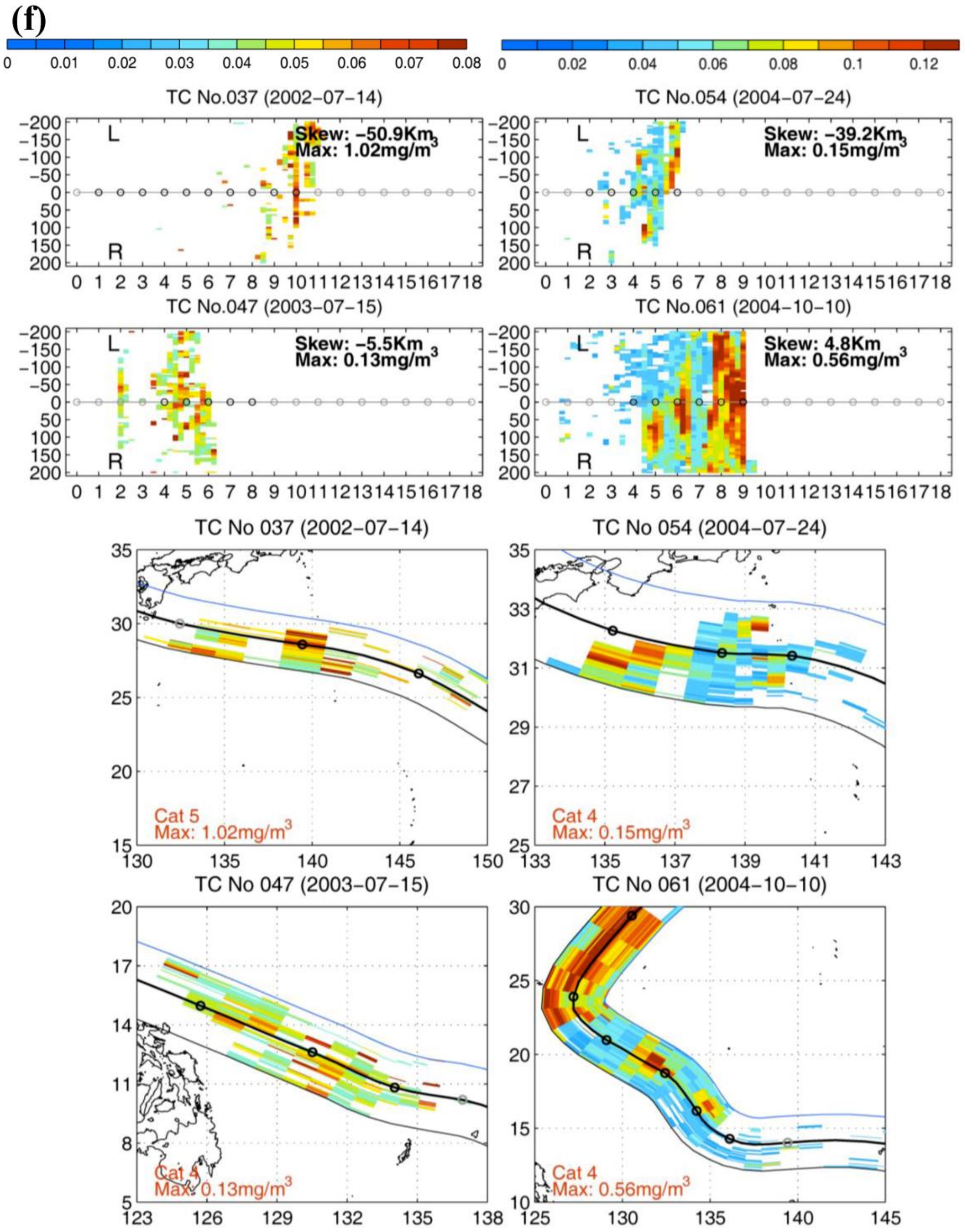

Fig.S2f Anecdotal examples of TC-induced Chl-a (anomalies from climatology, mg m^{-3}; t_{comp} = 10days) blooming, showing how the transformed along-track and cross-track maps (top 4 panels) look like when plotted on the geographic maps (bottom 4 panels). In each panel, black (grey) circles along the TC track denote daily positions when the TC becomes Category#1 and above (tropical storm). Maximum TC-Category, maximum Chl-a anomaly and skewness (Skew) of the composite are also displayed. (Maps were plotted using MATLAB Version#R2012a (7.14.0.739) 64-bit (glnxa64) http://www.mathworks.com/support/compilers/R2012a/glnxa64.html).

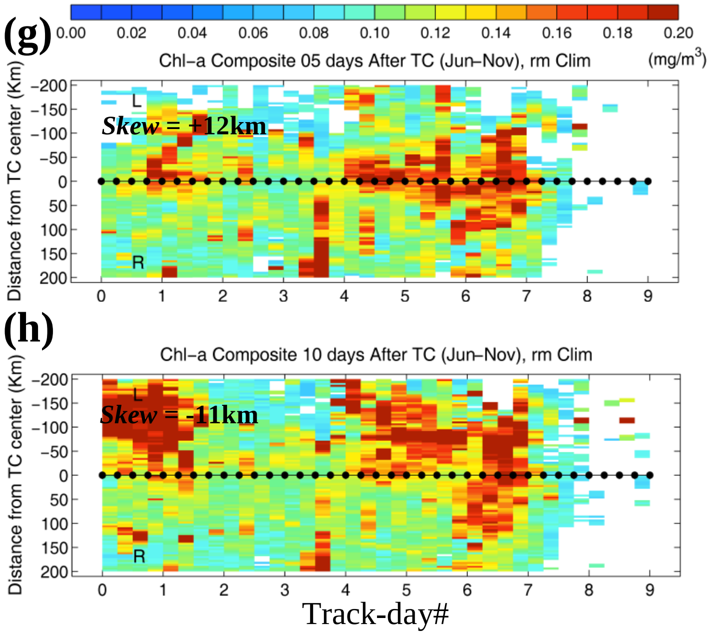

Fig.S2g,h Same as Fig.2a,b in the text: i.e. along- and cross-track composites of Chl-a anomaly for t_{comp} = 5 (g) & 10 (h) days, except that a threshold of 2×StD (= 0.074 mg m^{-3}) - i.e. Chl-a values which are less than 2×StD are omitted when calculating the composite. The effect is that fewer TCs (about 30) with large blooms or more complete data are included in this composite.

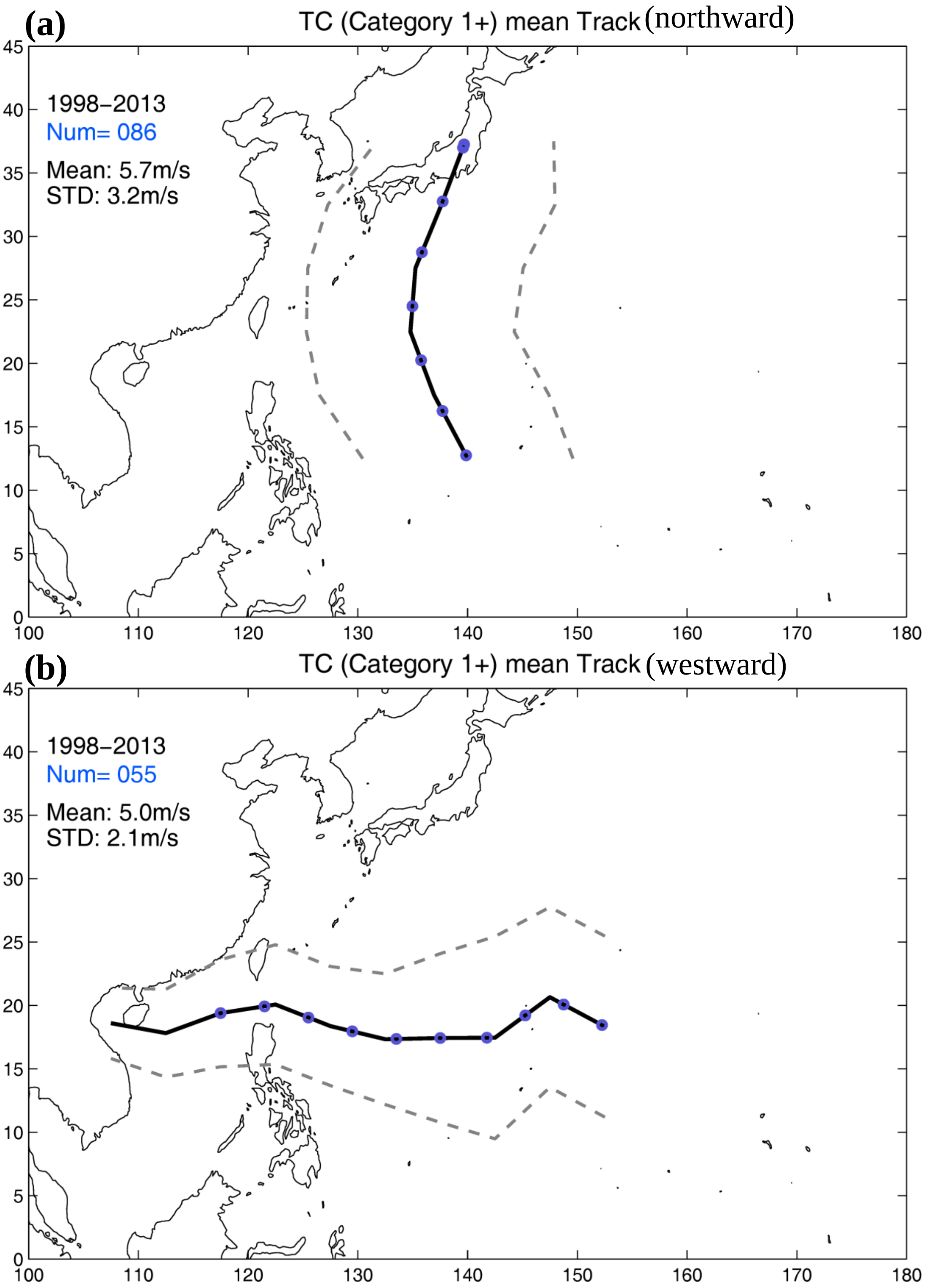

Fig.S3a,b Mean and ±1StD tracks for (a) northward and (b) westward translating groups of typhoons from 1998 to 2013 which are analyzed in this study. Dots denote mean daily positions of the typhoon center. (Maps were plotted using MATLAB Version#R2012a (7.14.0.739) 64-bit (glnxa64) http://www.mathworks.com/support/compilers/R2012a/glnxa64.html).

(c)

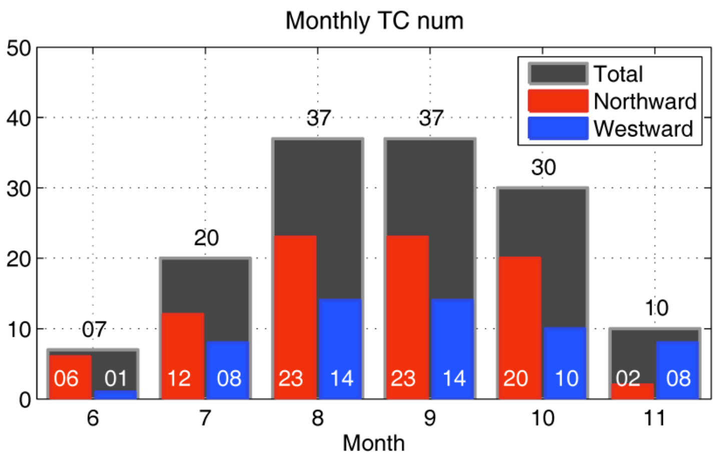

(d)

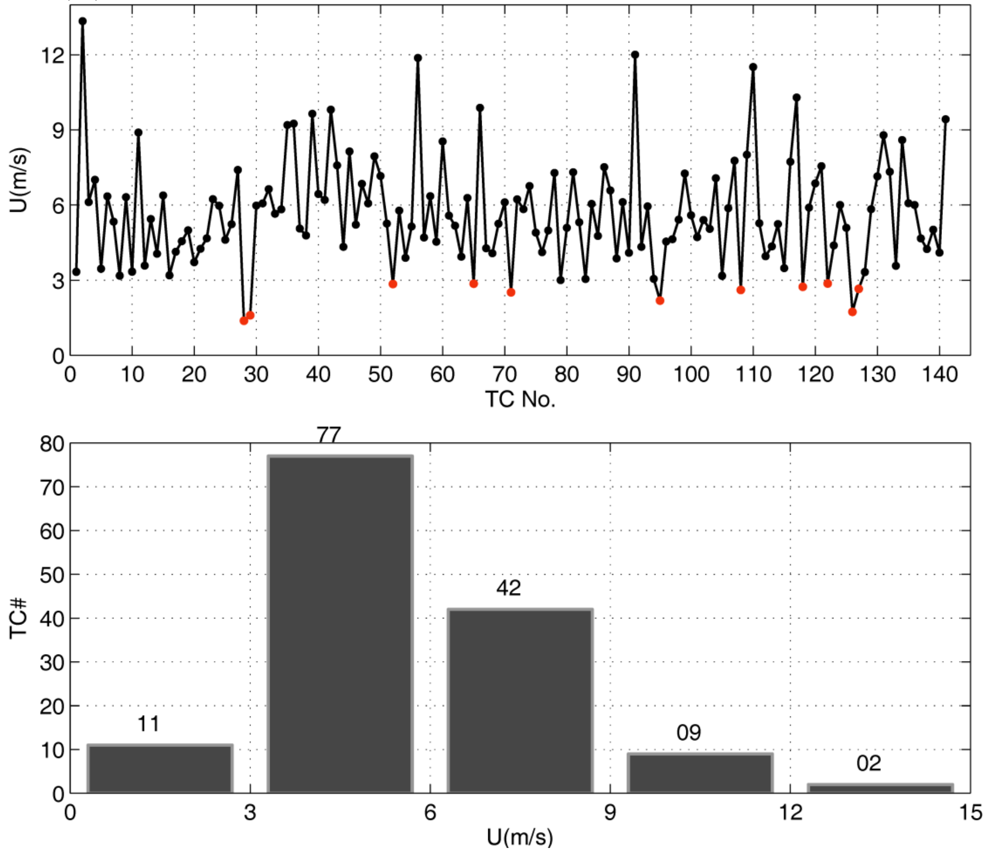

Fig.S3c,d (c) Monthly distributions of the total number of TCs and their partitions into northward and westward tracks as shown in Fig.S3a,b. (d) Averaged translation speeds of TCs.

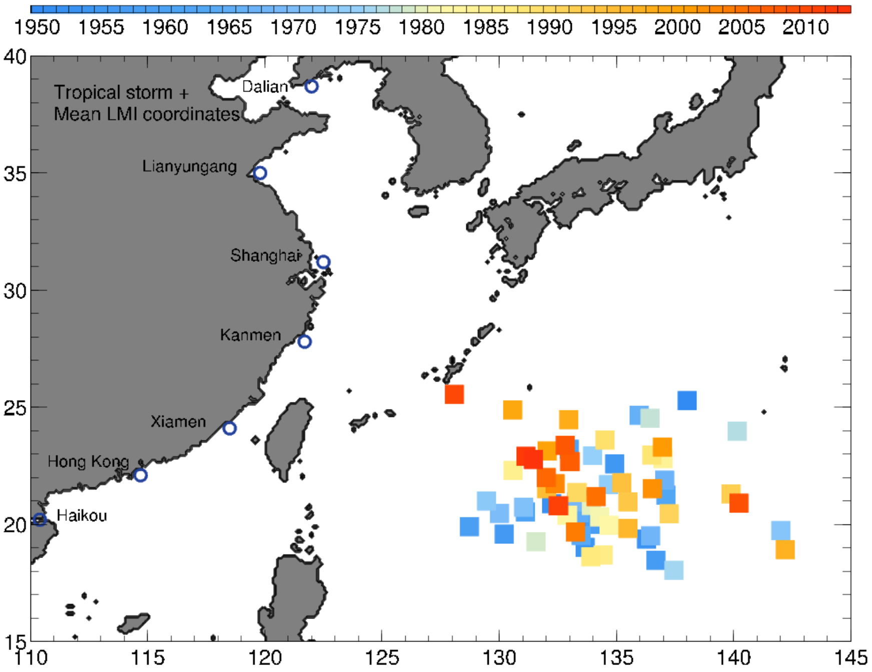

Fig.S4 Mean locations of TCs' life-time maximum wind speeds from 1950-2013, from the IBTrACS dataset, showing that the TCs are most intense when they reach near 20~25°N and west of 140°E. (Plot courtesy of Simon Chao). (Maps were plotted using MATLAB Version#R2012a (7.14.0.739) 64-bit (glnxa64) http://www.mathworks.com/support/compilers/R2012a/glnxa64.html).

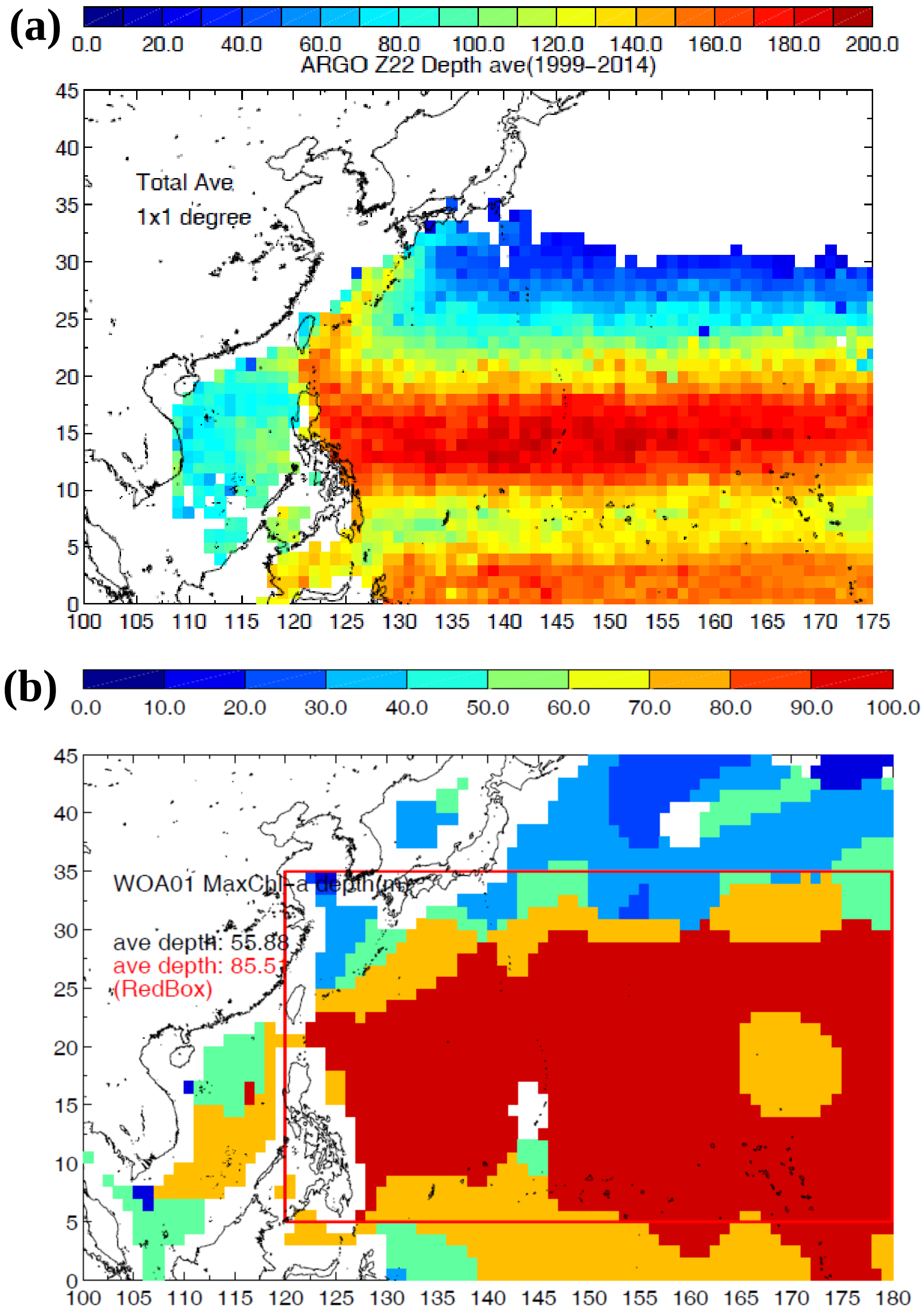

Fig.S5 Upper-layer depth as measured by the depth of the 22°C isotherm Z_{22} (m) calculated from the ARGO data (upper panel), and the depth of subsurface chlorophyll maxima from the WOA data (lower panel). (Maps were plotted using MATLAB Version#R2012a (7.14.0.739) 64-bit (glnxa64) http://www.mathworks.com/support/compilers/R2012a/glnxa64.html).

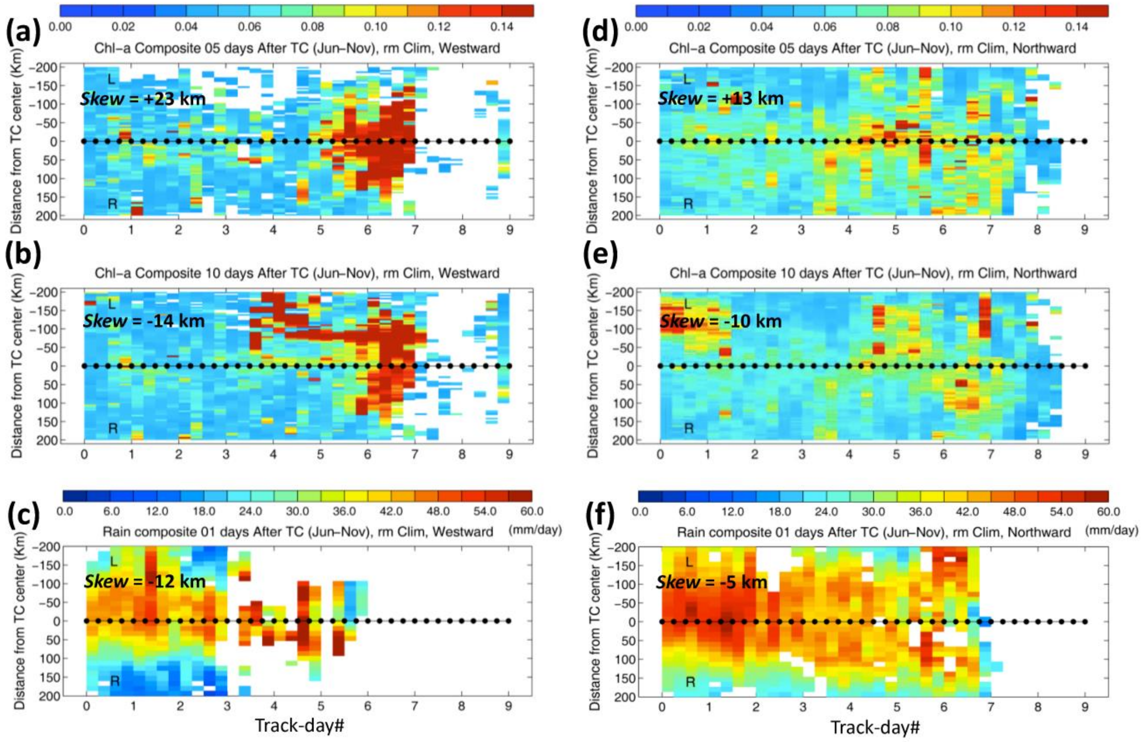

Fig.S6 Along- and cross-track composites for westward (a,b,c) and northward (d,e,f) typhoon tracks (Fig.1d), of Chl-a anomalies for t_{comp} = 5 (a & d) and 10 (b & e) days, and of 1-day composites of rainfall anomalies (c & f). The x-axis is along-track from 0 to 9 days and the y-axis is across-track ±200 km to the right (R) and left (L) of the track. Values are shown only if the anomaly exceeds the seasonal standard deviation. "*Skew*" is positive (negative) if composite is rightward- (leftward-) asymmetric.

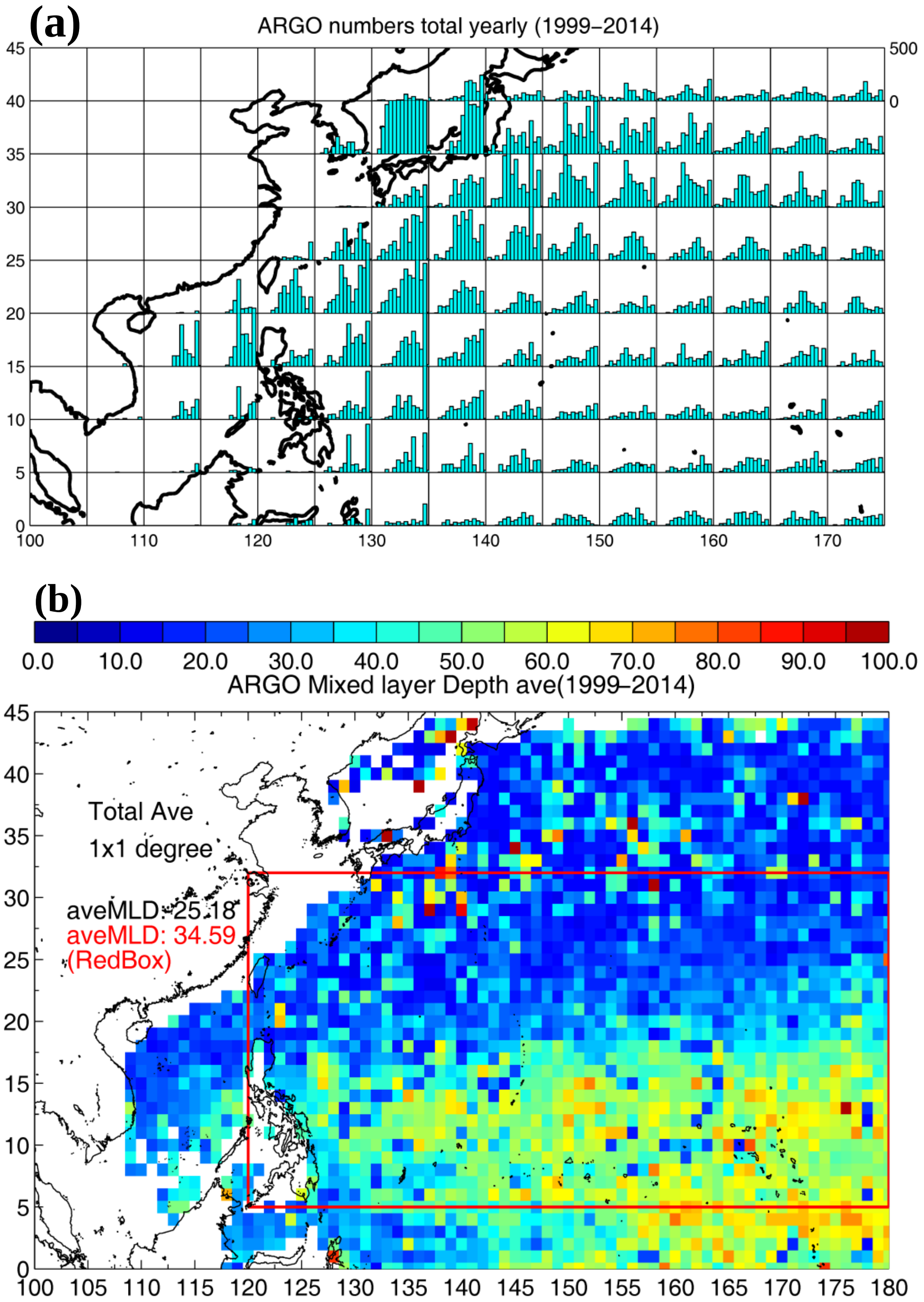

Fig.S7 (a) The number of ARGO floats inside every 5° x5^o grid. Each bar indicates each year from 1999 to 2014. (b) The averaged mixed layer depth (m) from 1999 to2014 with study area marked by the red box. The mean MLD is 25.18m for the entire domain and 34.59 for the study area. (Maps were plotted using MATLAB Version#R2012a (7.14.0.739) 64-bit (glnxa64) http://www.mathworks.com/support/compilers/R2012a/glnxa64.html).

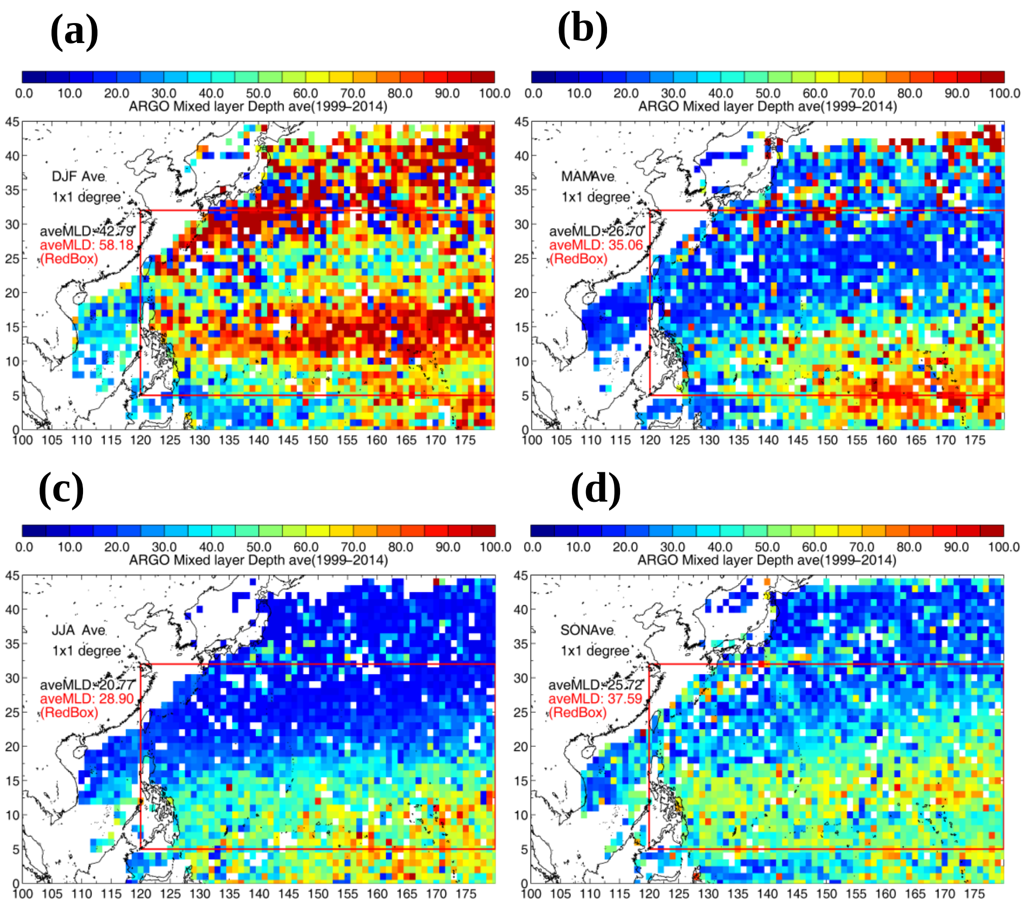

Fig.S8 Averaged MLD (m) for 4 seasons, (a) Dec, Jan and Feb; (b) Mar, Apr and May; (c) Jun, Jul and Aug; (d) Sep, Oct and Nov. (Maps were plotted using MATLAB Version#R2012a (7.14.0.739) 64-bit (glnxa64) http://www.mathworks.com/support/compilers/R2012a/glnxa64.html).

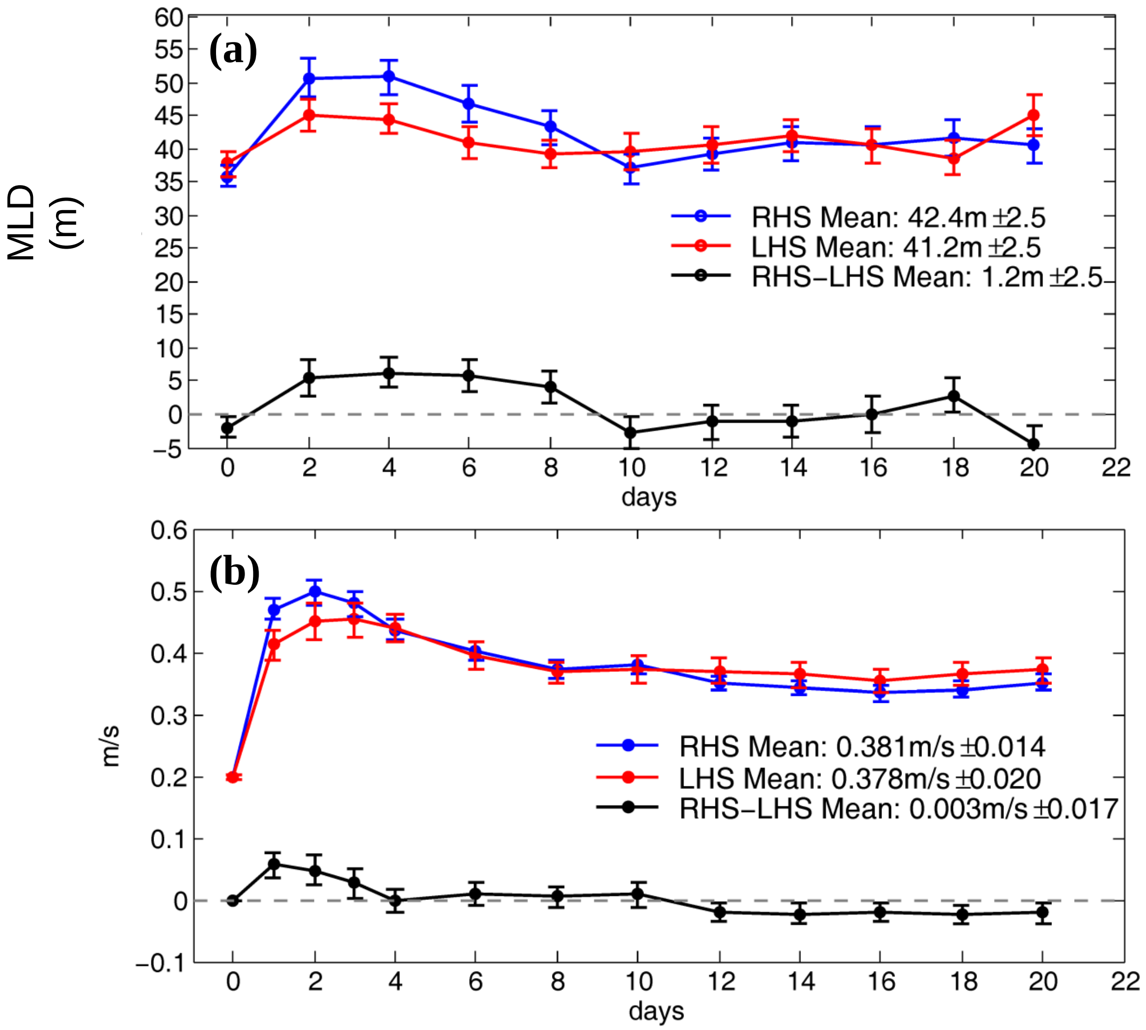

Fig.S9 (a) Mixed Layer depth (MLD; m) calculated from composites of all ARGO temperature profiles, and (b) inertial current speeds (m s^{-1}) calculated from composites of all surface drifters averaged on the right hand side (RHS) and left hand side (LHS) of TCs from 1999-2013, and plotted as a function of days (from 0 to 20 days) after the passages of the TCs. Bars indicate the standard errors of the composites. Note that the MLD relaxes to its background value after about 8 days, in general agreement with Foltz et al [ref.16].

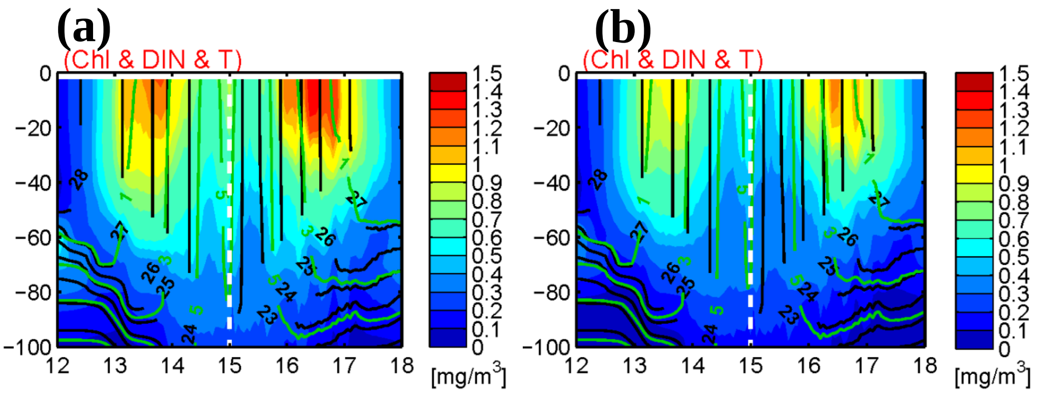

Fig.S10. The Huang and Oey's [ref.6] time-dependent one-dimensional (z-only) model (a) was repeated without the initial subsurface Chl-a maximum (b), i.e. the Chl-a is initially uniform = 0.12 mg m^{-3}); see Fig.2 of Huang and Oey [ref.6]. The plots show Chl-a (shading), DIN (green contours) and T (black contours) as a function of y (latitude) from 12°-18°N and z from -100 m to 0, approximately 5 days after the passage of a TC. The TC center is indicated by the vertical dashed line at y = 15°N (f=3.7746×10^{-5} s^{-1}). The view is westward in the direction of the translating TC, and variables have been zonally averaged from 110°-120°E. Panel (a) demonstrates how vertical mixing alone can produce stronger phytoplankton bloom on the right side of the TC centered at 15°N. In this case, right-side resonance generates stronger mixing which entrains more Chl-a from the subsurface chlorophyll-maximum to the surface, as well as subsurface DIN to the euphotic surface layer which then promotes phytoplankton growth. The two processes can be approximately separated; panel (b) shows the contribution from DIN alone without the subsurface chlorophyll-maximum, showing a weaker surface bloom. On the other hand, it shows that even in the absence of a subsurface chlorophyll-maximum, the larger amount of nutrient entrained to the surface produces more bloom on the right side.

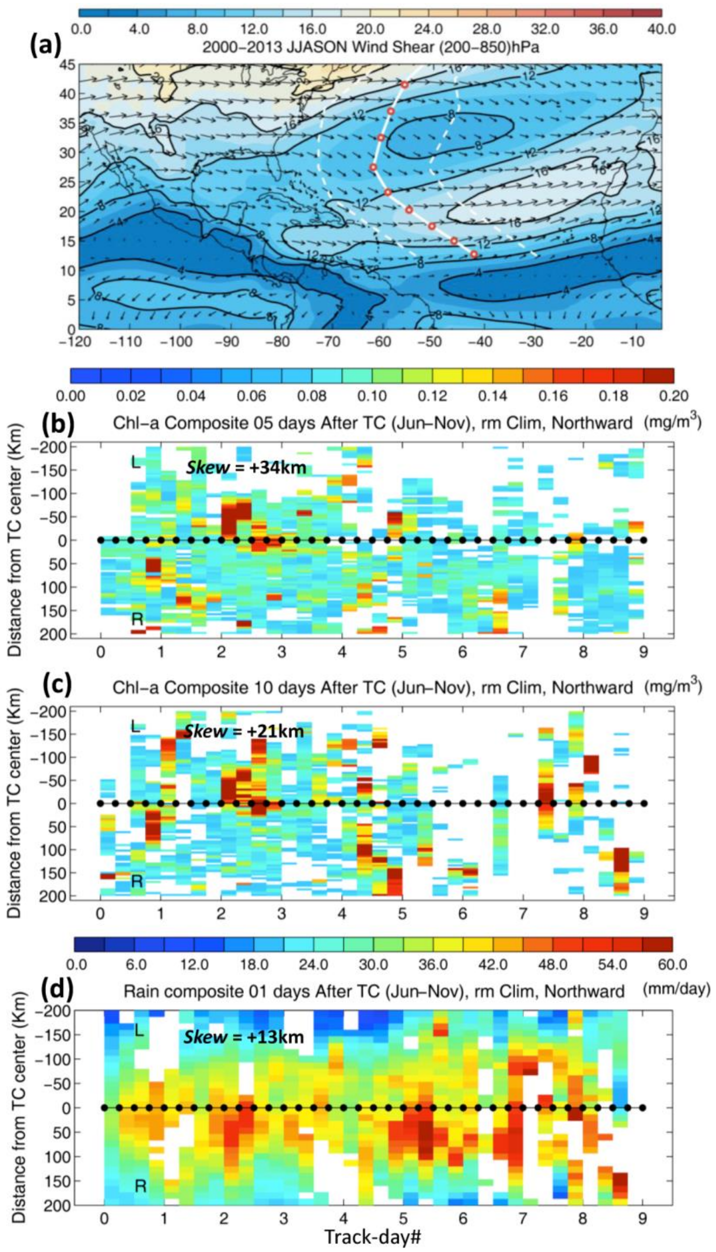

Fig.S11 (a) Climatological environmental vertical wind shear ($\mathbf{V}_s$ = wind at 200hPa – wind at 850hPa; color with vectors) in the North Atlantic. White lines are mean hurricane tracks with corresponding ±1StD in dashed lines. (b & c) Along- and cross-track composites of Chl-a anomaly for t_{comp} = 5 & 10 days and (d) of rainfall for t_{comp} = 1 day. The x-axis is along-track in "Track-day#" and y-axis is cross-track ±200 km to the right (R) and left (L) of the track. "*Skew*" is positive (negative) if composite is rightward- (leftward-) asymmetric. Sixty-six percent of hurricanes took the northward re-curving paths as shown in (a), while the remaining 34% continued westward into the Caribbean Sea and Gulf of Mexico. We excluded the westward tracks as these hurricanes cross lands and interact with orography, and rainfall pattern is not likely to cannot be simply inferred from wind shear alone. However, their composites also show rightward asymmetry in both Chl-a and rainfall (not shown), and including them in the composites shown here only has minor effects on the patterns. (Map in (a) was plotted using MATLAB Version#R2012a (7.14.0.739) 64-bit (glnxa64) http://www.mathworks.com/support/compilers/R2012a/glnxa64.html).
